# Human paternal and maternal demographic histories: insights from high-resolution Y chromosome and mtDNA sequences

**DOI:** 10.1186/2041-2223-5-13

**Published:** 2014-09-24

**Authors:** Sebastian Lippold, Hongyang Xu, Albert Ko, Mingkun Li, Gabriel Renaud, Anne Butthof, Roland Schröder, Mark Stoneking

**Affiliations:** 1 Department of Evolutionary Genetics, Max Planck Institute for Evolutionary Anthropology, Deutscher Platz 6, Leipzig D04103, Germany; 2 Department of Computational Genetics, CAS-MPG Partner Institute for Computational Biology, Shanghai 200031, China; 3 Present address: Fondation Mérieux, 17 rue Bourgelat, Lyon 69002, France; 4 Present address: Institute of Biochemistry, Faculty of Medicine, University of Leipzig, Leipzig D04103, Germany

**Keywords:** Population genetics, Population size, Simulations, HGDP

## Abstract

**Background:**

Comparisons of maternally-inherited mitochondrial DNA (mtDNA) and paternally-inherited non-recombining Y chromosome (NRY) variation have provided important insights into the impact of sex-biased processes (such as migration, residence pattern, and so on) on human genetic variation. However, such comparisons have been limited by the different molecular methods typically used to assay mtDNA and NRY variation (for example, sequencing hypervariable segments of the control region for mtDNA *vs.* genotyping SNPs and/or STR loci for the NRY). Here, we report a simple capture array method to enrich Illumina sequencing libraries for approximately 500 kb of NRY sequence, which we use to generate NRY sequences from 623 males from 51 populations in the CEPH Human Genome Diversity Panel (HGDP). We also obtained complete mtDNA genome sequences from the same individuals, allowing us to compare maternal and paternal histories free of any ascertainment bias.

**Results:**

We identified 2,228 SNPs in the NRY sequences and 2,163 SNPs in the mtDNA sequences. Our results confirm the controversial assertion that genetic differences between human populations on a global scale are bigger for the NRY than for mtDNA, although the differences are not as large as previously suggested. More importantly, we find substantial regional variation in patterns of mtDNA *versus* NRY variation. Model-based simulations indicate very small ancestral effective population sizes (<100) for the out-of-Africa migration as well as for many human populations. We also find that the ratio of female effective population size to male effective population size (N_f_/N_m_) has been greater than one throughout the history of modern humans, and has recently increased due to faster growth in N_f_ than N_m_.

**Conclusions:**

The NRY and mtDNA sequences provide new insights into the paternal and maternal histories of human populations, and the methods we introduce here should be widely applicable for further such studies.

## Background

Comparisons of mtDNA and NRY variation have provided numerous important insights into the maternal and paternal histories of human populations [1-3]. However, such comparisons are limited by methodological differences in how mtDNA and NRY variation have been typically assayed. MtDNA variation is usually investigated by sequencing hypervariable segments of the control region, (or, increasingly, via complete mtDNA genome sequences), while human NRY variation is routinely assayed by genotyping SNPs of interest, often in combination with short tandem repeat (STR) loci. Nevertheless, NRY SNP typing has several drawbacks due to the ascertainment bias inherent in the selection of SNPs [1,4,5]. This ascertainment bias complicates many analyses of interest, such as dating the age of the NRY ancestor or particular divergence events in the NRY phylogeny, as well as demographic inferences such as population size changes [4]. Moreover, the difference in molecular methods used to assay NRY *versus* mtDNA variation can complicate the interpretation of differences between patterns of NRY and mtDNA variation. For example, the seminal finding that NRY differences are bigger than mtDNA differences among global populations of humans, and that this is due to a higher rate of female than male migration due to patrilocality [6], may instead reflect methodological differences in how mtDNA *versus* NRY variation was assayed in that study [7].

Another fundamental question concerns whether or not male and female effective population sizes have been the same over time. Attempts to address this question using the ratio of X chromosome to autosomal DNA diversity have come up with conflicting answers [8,9], which may in part reflect the use of different methods that capture information about effective population size at different times in the past [10]. Moreover, the ratio of X to autosome diversity varies along the X chromosome, depending how far polymorphic sites are from genes [11-13], indicating a potential role for selection in distorting effective population size estimates from comparisons of X chromosome to autosomal DNA diversity. These and other fundamental aspects of human maternal and paternal demographic history remain unanswered.

Recently, analyses have been carried out of NRY sequences obtained as part of whole genome sequencing projects [14-16]. While these studies provide very detailed insights into the NRY phylogeny, they are nonetheless limited by the expense of whole genome sequencing, which precludes comprehensive global sampling. To allow for more accurate comparisons between mtDNA and NRY variation and to permit demographic inferences based on the NRY, we developed a capture-based array to enrich Illumina sequencing libraries for approximately 500 kb of NRY sequence. We used this approach to obtain NRY sequences from 623 males from 51 globally-distributed populations, and we also obtained complete mtDNA genome sequences from the same individuals, allowing us to investigate and directly compare the paternal and maternal relationships of global human populations in unprecedented detail.

## Methods

### Samples and sequencing library preparation

The samples consist of 623 males (Additional file 1: Table S1) from the CEPH Human Genome Diversity Panel (HGDP) [17]. The samples were taken from the subset ‘H952’, which excludes atypical, duplicated, and closely-related samples [18]. Approximately 200 ng of genomic DNA from each sample was sheared by sonication using a Bioruptor system (Diogenode) and used to construct an Illumina Sequencing library with a specific double-index as described previously [19]. The libraries were then enriched separately for NRY and mtDNA sequences as described below.

### Y-chromosome capture array design

We targeted unique regions on the NRY that are free of repeats and to which the typically short next-generation sequencing reads could be mapped with high confidence. We used the UCSC table browser [20] and the February 2009 (GRCh37/hg19) assembly and applied the following filter criteria. First, from the group ‘variation and repeats’, sequence regions annotated in the following tracks were removed: Interrupted Repeats, RepeatMasker, Simple Repeats, and Segmental Duplications. Next, we used the ‘mapability’ table ‘CRG Align 75’ from the group ‘mapping and sequencing tracks’ to identify and remove regions with mapability scores below 1. We then removed regions of less than 500 bp in order to reduce the number of fragments and thereby the number of fragment ends, which have low probe densities. We also removed 15mers that occurred more than 100 times in the hg19 genome assembly, as described previously [21], which resulted in splitting some target regions into sub-regions that were less than 500 bp. The final result was a total of approximately 500 kb of unique NRY sequence, distributed among 655 target regions ranging from 61 bp to 3.9 kb (Additional file 2: Table S2). These regions were then used to design a custom array (SureSelect 1 M capture array, Agilent) with 60 nt probes that were printed twice with a tiling density of 1 bp.

### NRY enrichment

Up to 60 barcoded libraries were pooled in equimolar ratio. The library mix was enriched for target NRY regions by hybridization-capture on the custom designed array following the protocol described previously [22]. After enrichment the library-pool was quantified by qPCR and then amplified to a total of approximately 10^12^ molecules. The final concentration and length distribution was measured on an Agilent DNA 100 microchip, and 10 nmol of the amplified library pool was used for sequencing. Each pool, consisting of 48 to 60 samples, was sequenced on a Solexa GAII lane using a paired end 75 cycle run plus two 7 nt index reads.

### MtDNA enrichment

Up to 94 libraries were pooled in equimolar ratio and the library pool was enriched for mtDNA sequences by an in-solution hybridization capture method [23]. The hybridization eluate was measured by qPCR and then amplified to produce a final concentration of 10 nmol. Up to 200 samples were sequenced on a Solexa GAII lane using a paired end 75 cycle run, plus two 7 nt index reads.

### Data processing

In each Solexa GAII lane, 1% PhiX174 phage DNA was spiked in and used as a training set to estimate base quality scores with the IBIS base-caller [24]. Reads with more than five bases having a PHRED scaled quality score below Q15 were discarded, as were reads having a single base quality in the index read (7 nt) score below Q10. Reads with no mismatches to the expected double index sequences were assigned to each individual sample library.

For the NRY-enriched data, reads were mapped to the human reference genome (GRCh37) using default settings with BWA v0.5.10 [25]. We mapped to the whole genome rather than just the target region, in order to identify reads that might, with equal probability, map to another position in the genome. The bam files containing the mapping information and reads were processed with samtools v0.1.18 [26]. We used Picard 1.42 to mark duplicates, based on the start and end coordinates of the read pairs. The final SNP call was done on all samples simultaneously using the UnifiedGenotyper from the GATK v2.0-35 package [27] and the following options: --output_mode EMIT_ALL_CONFIDENT_SITES, --genotype_likelihoods_model SNP, --min_base_quality_score 20 and --heterozygosity 0.0000000001. The result was stored in a VCF file containing information for each callable site of the target region, and a second VCF file was created that contained only the variable positions among the 623 samples. For each sample at each variable position the PL scores were calculated with samtools [26]; PL scores are normalized, PHRED-scaled likelihoods for the three genotypes (0/0, 0/1, 1/1) and are based on, among other things, coverage, base quality, and mapping quality. Positions that showed a difference in the PL score of less than 30 between homozygote reference (0/0) and homozygote alternative (1/1) were called an ‘N’ in that sample, as were positions where heterozygote calls (0/1) either had a higher PL score than the most likely homozygous genotype, or differed by less than 30 from the most likely homozygous genotype. Note that a PL score of 30 between genotype 0/0 and 1/1 means that the former is 1,000 times more likely than the latter, for example, the genotype-calling error rate is expected to be less than 1 in 1,000. Sites where more than two bases were called (that is, multi-allelic sites) were also removed.

For the mtDNA-enriched data, reads were mapped to the revised mtDNA reference sequence (GenBank number: NC_012920) using the software MIA [28]. The consensus sequences were aligned using MUSCLE v3.8.31 [29] (cmd line: muscle -maxiters 1 -diags mt_623seq.fasta mt_623seq.aln), and haplogroups were called with the HaploGrep software [30].

### Imputation for the NRY

After quality filtering, there were 2,276 variable sites in the NRY sequences, with a total of 2.54% of the individual genotypes at variable positions scored as ‘N’ (that is, as missing data; the number of missing sites per individual ranged from 9 to 1,173, with an average of 122 missing sites per individual). Since missing data can influence the results of some analyses, we took advantage of the fact that the NRY target regions are completely linked with no recombination to impute missing data as follows. First, all sites with no missing data (605 sites) were used as the reference set to define haplotypes and calculate the number of differences between each haplotype. Sites with missing data were then imputed, beginning with the site with the smallest amount of missing data and proceeding sequentially. For each haplotype with missing data for that site, the missing base was imputed as the allele present in the reference haplotype that had the fewest differences (based on the sites with no missing data). After imputation was finished for that site, it was added to the reference set, and the procedure continued for the next site with the smallest amount of missing data.

As a check on the accuracy of the imputation, we randomly deleted 2.54% of the known alleles, following the distribution of missing alleles in the full dataset, thereby creating an artificial dataset with a similar distribution of missing alleles as in the observed dataset. We then imputed the missing data according to the above procedure and compared the imputed alleles to the true alleles; this procedure was carried out 1,000 times. The imputed allele matched the true allele in 99.1% of the comparisons, indicating that the imputation procedure is quite accurate.

### Recurrent NRY mutations

We expect the majority of the NRY SNPs to have mutated only once, as recurrent mutations in the known NRY phylogeny are quite rare [31,32]. Therefore, as a further quality control measure, we investigated the NRY data for recurrent mutations by constructing a maximum parsimony tree for the 2,276 SNPs using programs in PHYLIP. We then estimated the number of mutations at each SNP, and removed 48 SNPs that had mutated more than twice, and only in terminal branches, as these are likely to reflect sequencing errors. The final dataset contains 2,228 SNPs.

### Data analysis

Basic summary statistics (haplotype diversity, mean number of pairwise differences, nucleotide diversity, Tajima’s D value and theta(S)) were calculated using Arlequin v3.5.1.3 [33]. Arlequin was further used to estimate pairwise Φ_ST_ values and for Analysis of Molecular Variance (AMOVA). The observed ratio of the mean pairwise differences (mpd) for the NRY *versus* mtDNA was calculated as mpd_NRY_/mpd_mt_. In order to detect group-specific deviations from the mean distribution of the mpd ratio in the dataset, we carried out a resampling approach. For each group sample size (N_group_) we chose randomly N_group_ individuals (out of 623) and calculated the mpd ratio using the dist.dna command from the APE package [34] in R. This was repeated 10,000 times for each N_group_ sample size to obtain the distribution of resampled mpd ratios.

Divergence times in the NRY and mtDNA phylogenies were estimated using a Bayesian approach implemented in BEAST v1.6.2 [35]. For the mtDNA genome sequences we divided the alignment into two partitions consisting of the coding and non-coding regions, respectively. For both partitions we estimated the best fitting substitution model using jModeltest [36] and the mutation rates estimated previously [37]. These rates were calibrated by a combination of chimpanzee-human divergence and archaeological colonization dates, take into account time-dependency in the molecular clock, and are in the range of recent estimates of the mtDNA mutation rate [15,38,39]. For the non-coding region we used the GTR + I + G substitution model and a mutation rate of 9.883 × 10^−8^ substitutions/site/year, while for the coding region we used the TrN + I + G model and a mutation rate of 1.708 × 10^−8^ substitutions/site/year. A strict clock and a constant size coalescence model were used, and the MCMC was run for 10 million steps with sampling from the posterior every 2,000 steps. The MCMC was run on five independent chains in parallel. After careful inspection of the log files in Tracer, the tree files of the five runs were merged after discarding the first 2,500 trees (50%) of each run as burn-in. A consensus tree was built from the merged trees using TreeAnnotator, and the consensus tree showing the divergence times for each node was visualized with FigTree.

For the NRY sequences the same procedure was used, but modified as only variable sites were included in the BEAST analysis in order to reduce the computational time. The substitution model used was HKY without I + G, and the substitution rate was multiplied by the number of callable sites (501,108 sites) divided by the number of variable sites (2,228 sites). As there is uncertainty regarding the mutation rate, we ran the analysis twice, with a ‘fast’ rate [40] of 1.00 × 10^−9^ substitutions/site/year (transformed to 2.25 × 10^−7^) and with a ‘slow’ rate [41] of 6.17 × 10^−10^ substitutions/site/year (transformed to 1.39 × 10^−7^).

Bayesian skyline plots [42] were used to estimate population size change through time, using the same mutation rates and substitution models described above. The piecewise-linear Skyline coalescence model was chosen and the number of groups (bins) was set to half the sample size per group with a maximum of 20. A single MCMC chain was ran for 30 million steps and sampled every 3,000 steps from the posterior. The log file was inspected in Tracer for convergence of the chain and ESS values and the Bayesian Skyline Reconstruction was run.

### Simulations

**Figure 1:**
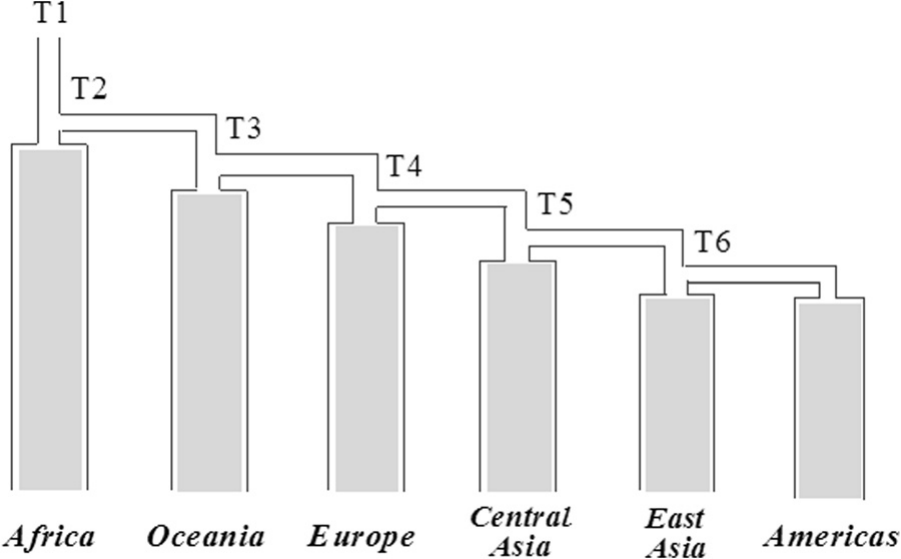
**The model of population history used in simulations.** We assumed a single out-of-Africa migration and further population divergence events (see text for further details). The model begins with the ancestral population in Africa (at time T1), a single out-of-Africa migration (T2), the first split between Oceania and Eurasia (T3), then Europe and Asia (T4), followed by Central and East Asia (T5), and finally between East Asia and the Americas (T6). We also required T2 to be greater than T3. The model assumes no migration between regions following divergence; in support of this assumption, there is very little sequence sharing between regions. We do allow changes in population size. This model was first used to estimate divergence times with combined mtDNA and NRY sequences, then the model and estimated mean divergence times were used in separate simulations of the mtDNA and NRY sequences to estimate ancestral and current N_f_ and N_m_.

We used a simulation-based approach to estimate current and ancestral effective population sizes, based on either mtDNA or NRY sequences, for each regional grouping of populations. We started with the model of population history shown in Figure 1, which consists of six geographic regions, and corresponds to a tree built from genome-wide SNP data from the HGDP populations [43], with the exception that Oceania branches off first among non-African populations in Figure 1 rather than directly from East Asia. This alternative placement of Oceania is in keeping with subsequent studies of genome-wide data that have tested various models and found strongest support for an early branching of Oceanian population [44-46]. The model includes 44 populations and 511 individuals; we excluded the Adygei, Uygur, Hazara, and all of the ME/NA populations as these exhibit high levels of admixture between the regional groups in genome-wide analyses [43,47]. We first simulated the combined mtDNA and NRY sequences with the fastsimcoal software [48] and used approximate Bayesian computation (ABC) [49] to estimate divergence times based on the combined dataset, with the same mtDNA mutation rate used in the BEAST analysis and an average of the fast and slow NRY mutation rates. We simulated 5,808,805 observations, which were log transformed via ABC linear regression [49] using the following statistics: polymorphic sites (S), pairwise differences (Pi), Tajima’s D, pairwise Φ_st_, and the variance components for an AMOVA based on two groups, Africa *versus* non-Africa (the latter consisting of the pooled data from the five non-African regional groups). We then used this history (Figure 1) and the mean divergence times based on the combined data in a further set of simulations to estimate from the mtDNA and NRY sequences the ancestral and current effective population sizes, for females and males, respectively, for each regional group of populations. We simulated 5,116,984 observations for the mtDNA sequences and 5,325,179 observations for the NRY sequences, and retained the top 1,000 simulations (tolerance of 0.03%) in each case for parameter estimation.

## Results

### NRY and mtDNA diversity

We obtained approximately 500 kb of NRY sequence from the 623 males in the HGDP, and complete mtDNA genome sequences from these 623 males plus an additional 329 females from the HGDP. The average coverage of the NRY sequences was 14.5X (range, 5X-37.5X, Additional file 3: Figure S1), while for the mtDNA genome sequences the average coverage was 640X (range, 46X-4123X, Additional file 3: Figure S1). After quality-filtering, imputation, and removal of sites with a high number of recurrent mutations, there remained 2,228 SNPs in the NRY sequences. The mtDNA analyses here are restricted to the 623 males for which NRY sequences were obtained, for which there were 2,163 SNPs; results based on the mtDNA genome sequences from the entire set of HGDP samples (952 individuals) did not differ from those based on the subset of 623 males (for example, Additional file 3: Figure S2). More details about the results from each individual, including mtDNA and NRY haplogroups, are provided in Additional file 1: Table S1. The mtDNA sequences have been deposited in Genbank with accession numbers KF450814-KF451871. A datafile with the alleles at each of the NRY SNPs in each sample has been provided to the CEPH-HGDP and additionally is available from the authors. The NRY raw sequencing data are in the European Nucleotide Archive with the study accession number PRJEB4417 (sample accession numbers ERS333252-ERS333873).

Basic summary statistics for the mtDNA and NRY diversity in each population are provided in Additional file 3: Table S3. As the sample sizes for many of the individual populations are quite small, for most subsequent analyses we grouped the populations into the following regions (based on analyses of genome-wide SNP data [43,47]): Africa, America, Central Asia, East Asia, Europe, Middle East/North Africa (ME/NA), and Oceania (the regional affiliation for each population is in Additional file 1: Table S1). The Adygei, Hazara, and Uygur were excluded from these groupings as they show evidence of substantial admixture between these regional groups [43,47]. We stress that the use of regional names is a convenience to refer to these groupings of these specific populations, and should not be taken to represent the entirety of the regions (for example, ‘Africa’ refers to the results based on the analysis of the combined African HGDP samples, not to Africa in general).

**Figure 2:**
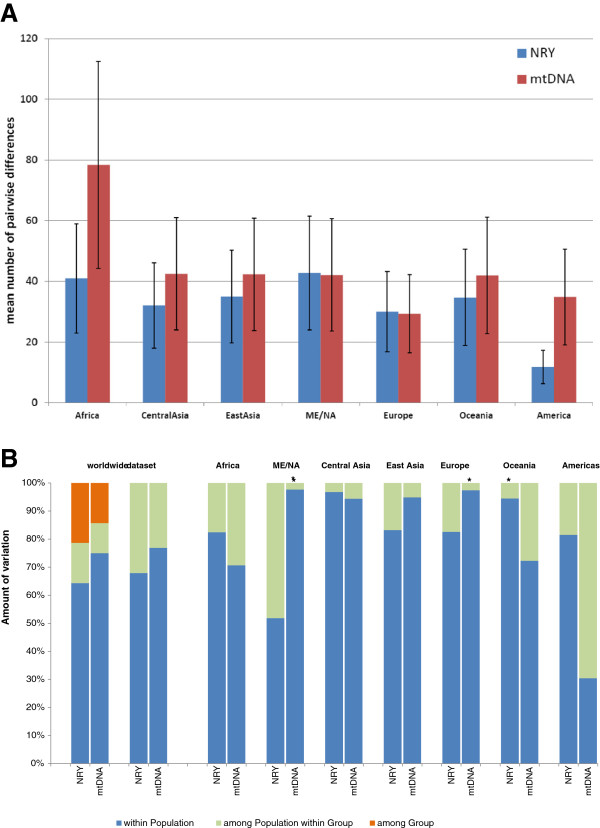
**Diversity and AMOVA results. (A)** Mean number of pairwise differences (and SE bars) for the NRY and mtDNA sequences from each regional group. **(B)** AMOVA results for the entire worldwide dataset, and for each regional group of populations. Two comparisons are shown for the entire dataset; the left comparison includes regional groups as an additional hierarchical level, while the right one does not. * indicates that the among-population component of diversity does not differ significantly from zero (after Bonferroni adjustment of the *P* value for multiple comparisons).

Some basic summary statistics concerning mtDNA and NRY diversity for the regions are provided in Table 1. The π values we report are for the most part somewhat larger than reported in a previous study of eight Africans and eight Europeans [50], which is not unexpected given the much larger sampling in our study. Notably, we find substantial variation among geographic regions in amounts of mtDNA *versus* NRY diversity; this is shown further in the comparison of the mean number of pairwise differences (mpd) for mtDNA and the NRY (Figure 2A). The mtDNA mpd for Africa is about twice that for other regions, while the NRY mpd is greatest in the Middle East/North Africa region, and only slightly greater in Africa than in the other regions (with the exception of the Americas, which show substantially lower NRY diversity). Overall, there are striking differences in the ratio of NRY:mtDNA mpd (Table 1), with Africa, Central Asia, and the Americas having significantly less NRY diversity relative to mtDNA diversity, compared to the other regional groups. Moreover, differences in relative levels of NRY:mtDNA diversity are also evident in the individual populations (Additional file 3: Table S3), although the small sample sizes indicate that the individual population results must be viewed cautiously.

**Table 1:** Summary statistics for regional groups

	**NRY**					**mtDNA**				
**Group**	**n**	**H**	**S**	**mpd ± SE**	**π ± SE**^**a**^	**H**	**S**	**mpd ± SE**	**π ± SE**^**b**^	**mpd ratio**
Africa	85	71	545	41.0 ± 18.0	80 ± 40	70	617	78.3 ± 34.0	47 ± 23	0.52^c^
Central Asia	146	106	524	32.1 ± 14.1	62 ± 31	131	833	42.4 ± 18.5	26 ± 12	0.76^c^
East Asia	162	141	709	35.0 ± 15.3	71 ± 36	156	899	42.3 ± 18.5	26 ± 12	0.83
ME/NA	75	47	301	42.7 ± 18.7	85 ± 40	71	618	42.0 ± 18.4	25 ± 12	1.02
Europe	79	68	350	30.0 ± 13.2	58 ± 31	78	432	29.3 ± 12.9	18 ± 9	1.02
Oceania	17	16	147	34.7 ± 15.9	71 ± 36	16	175	41.9 ± 19.2	25 ± 13	0.83
America	22	19	96	11.8 ± 5.5	22 ± 13	15	148	34.9 ± 15.8	21 ± 11	0.39^c^

^a^Multiply values by 10^−6^.

^b^Multiply values by 10^−4^.

^c^Group ratios that differ significantly (*P* <0.05) from the overall average ratio for the entire HGDP, based on random resampling of NRY and mtDNA sequences.

H, number of different haplotypes (sequences); mpd ratio, ratio of the mpd_NRY_/mpd_mtDNA_; n, sample size; S, number of polymorphic sites; mpd ± SE, mean number of pairwise differences ± standard error; π ± SE, nucleotide diversity ± standard error.

### NRY and mtDNA population differentiation

An outstanding question is whether or not there are differences in the relative amounts of between-population *versus* within-population diversity for mtDNA *versus* the NRY, as some studies have found much larger between-population differences for the NRY than for mtDNA [6] while others have not [7]. To address this question, we carried out an AMOVA; the results (Figure 2B) show that in the entire worldwide dataset, the between-population differences are indeed bigger for the NRY (approximately 36% of the variance) than for mtDNA (approximately 25% of the variance). However, there are substantial differences among the regional groups. The ME/NA, East Asia, and Europe regional groups follow the worldwide pattern in having bigger between-population differences for the NRY than for mtDNA. In contrast, Africa, Oceania, and the Americas have substantially bigger between-population differences for mtDNA than for the NRY, while for central Asia the between-population variation is virtually identical for the NRY and mtDNA. These regional differences likely reflect the influence of sex-biased migrations and admixture, as discussed in more detail below, and moreover indicate that focusing exclusively on the worldwide pattern of mtDNA *versus* NRY variation misses these important regional differences.

We also investigated the relationship between geography and genetic distance. Despite the small sample sizes at the population level, both mtDNA and NRY Φ_ST_ distances are significantly correlated with geographic distances between populations (Mantel tests with 1,000 replications: mtDNA, r = 0.41, *P* <0.001; NRY, r = 0.36, *P* = 0.002) as well as with each other (r = 0.23, *P* = 0.025). Thus, NRY and mtDNA divergence are both highly associated with geographic distances among populations.

### MtDNA and NRY phylogenies

**Figure 3:**
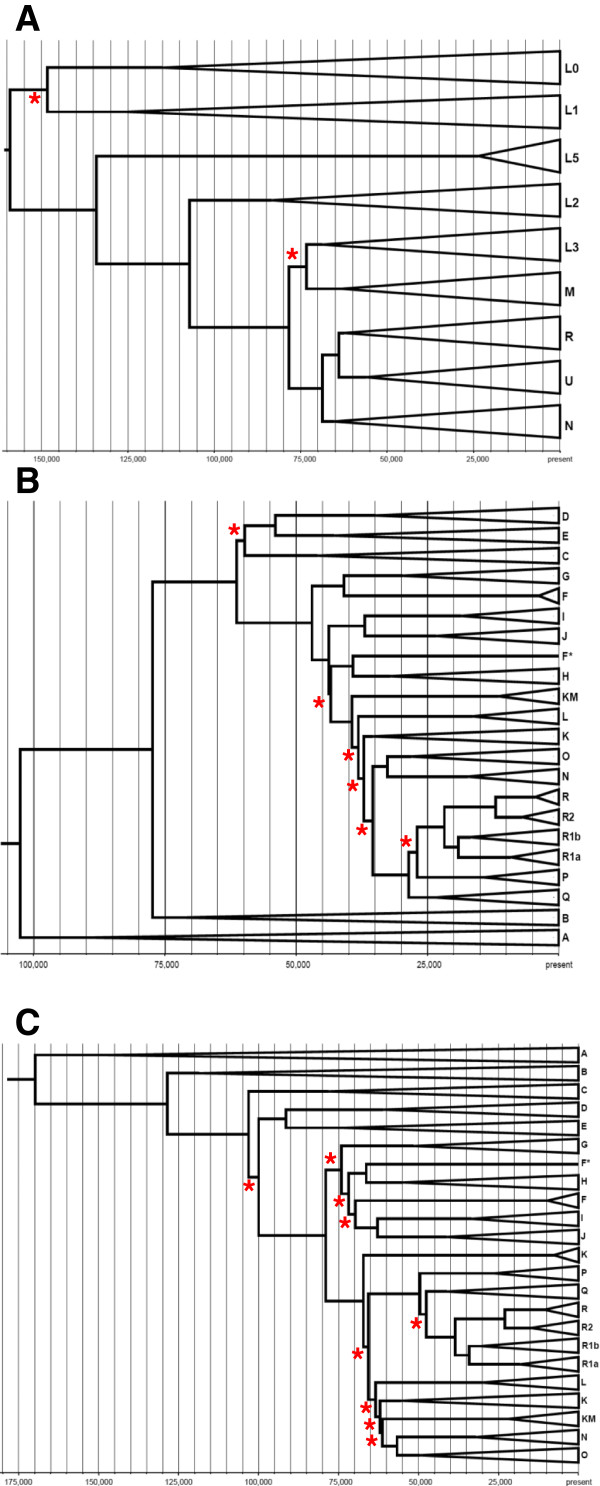
**Bayesian trees and divergence time estimates for mtDNA and NRY haplogroups. (A)** mtDNA haplogroups; **(B)** NRY haplogroups with the fast mutation rate; **(C)** NRY haplogroups with the slow mutation rate. Red asterisks denote nodes with low support values (<0.95). F* in the NRY trees indicates a sample that was assigned to haplogroup F by SNP genotyping, but does not fall with other haplogroup F samples. Some NRY haplogroup K samples formed a monophyletic clade (labelled K in the trees) while others fell with haplogroup M samples (labelled KM in the trees); see also Additional file 3: Figure S8.

Although the primary purpose of this study is to compare demographic insights from mtDNA and NRY sequences that were obtained free of the ascertainment bias inherent in haplogroup-based approaches, we recognize that there is also useful information in the haplogroups. In this section we therefore present some haplogroup-based results. We first used a Bayesian method to estimate the phylogeny and divergence times for both mtDNA and the NRY (Figure 3); for the latter, we used both a ‘fast’ mutation rate of 1 × 10^−9^/bp/year and a ‘slow’ mutation rate of 0.62 × 10^−9^/bp/year as there is currently much uncertainty regarding mutation rates [5,40,41,51,52]. The resulting phylogenies are in general consistent with the existing mtDNA and NRY phylogenies [31,53], although there are some discrepancies, for example, in the mtDNA tree (Figure 3A) L1 sequences group with L0 sequences rather than on the other side of the root, while additional discrepancies can be found in the NRY trees. However, all of these discrepancies involve nodes that have low support values (red asterisks in Figure 3) and hence low confidence; the nodes that have strong support values are all in agreement with the existing mtDNA and NRY phylogenies. The inability of the Bayesian analysis to completely resolve the phylogenies has two causes: for the mtDNA phylogeny, frequent back mutations and parallel mutations at some sites confounds the analysis; for the NRY phylogenies, some branches in the accepted phylogeny are supported by only a few SNP positions that are not included in our sequence data.

The age of the mtDNA ancestor is estimated to be about 160 thousand years ago (kya), and the ages of the non-African mtDNA lineages M and N are about 65 to 70 kya, in good agreement with previous estimates [54]. Our estimate for the age of the NRY ancestor is 103 kya based on the fast rate, and 165 kya based on the slow rate; however these estimates do not include the recently-discovered ‘A00’ lineage [41], which would result in much older ages for the NRY ancestor. The close agreement between the slow NRY ancestor age (165 kya) and the mtDNA ancestor age (160 kya) might be taken as evidence in favor of the slow NRY mutation rate. However, the slow NRY mutation rate gives an estimated age for the initial out-of-Africa divergence of about 100 kya, and an age for the divergence of Amerindian-specific haplogroup Q lineages of about 20 kya, while the fast rate gives corresponding estimates of about 60 kya for out-of-Africa and about 12.5 kya for Amerindian haplogroup Q lineages, in better agreement with the mtDNA and other evidence for these events [54-57]. Given the current uncertainty over mutation rate estimates, we have chosen to use either both estimates in further analyses (for example, Bayesian skyline plots) or an average of the fast and slow rates (for example, in simulation-based analyses); in Additional file 3: Table S4 we provide divergence time estimates and associated 95% credible intervals for the branching events shown in the phylogenies in Figure 3.

NRY and mtDNA haplogroup frequencies per population are shown in Additional file 3: Table S5 and Additional file 3: Table S6, respectively. The mtDNA haplogroups were called from the sequences determined here, while the NRY haplogroups were previously determined by SNP genotyping [58,59]. The NRY haplogroup information we provide is taken only from these published data; we did not infer haplogroups from the sequences, in order to have an independent comparison of the NRY tree with the haplogroups. The phylogenetic relationships for the NRY sequences are generally concordant with the SNP-genotyping results (with some exceptions, discussed in the legends to Figures S3 to S12 in Additional file 3). The haplogroup frequencies provide further insights into some of the different regional patterns of mtDNA *versus* NRY diversity noted previously. For example, the comparatively low diversity and smaller differences among populations for the NRY in Africa is due to the high frequency of NRY haplogroup E (55% to 100% in the non-Khoisan groups; Additional file 3: Table S5). This haplogroup is widespread in western Africa, and specific subhaplogroups of haplogroup E are associated with the Bantu expansion [59-61]. The comparatively low NRY diversity in the HGDP Africa regional group thus likely reflects a ‘homogenizing’ effect of the Bantu expansion. NRY haplogroup E is also of interest because it occurs in some European and ME/NA groups, at frequencies of up to 17%, as well as in a few individuals from Central Asia (Additional file 3: Table S5). Inspection of the phylogeny of haplogroup E sequences (Additional file 3: Figure S7) reveals that all of the European and most of the ME/NA haplogroup E sequences form a clade distinct from the African haplogroup E sequences, and the age of this clade is about 18 kya. Moreover, all of the European haplogroup E sequences fall into a subclade that is about 14 kya. These results may reflect a migration from North Africa to Europe suggested from analyses of genome-wide SNP data [62], and would thus provide a timeframe for this migration.

In Oceania, the bigger differences between populations for mtDNA than for the NRY (Figure 2B, Table 1) probably reflect the high frequency of mtDNA haplogroup B in just one of the two Oceania populations (75% in the Melanesian population *vs.* 0% in the Papuan population; Additional file 3: Table S6). MtDNA haplogroup B is associated with the Austronesian expansion [63-65]. By contrast, NRY haplogroups associated with the Austronesian expansion, such as haplogroup O [63,66,67] are absent in the HGDP Oceania populations (Additional file 3: Table S5). This contrast further testifies to the larger maternal than paternal impact of the Austronesian expansion on Oceanian populations [63,66-69].

In the Americas, there are dramatic differences in mtDNA haplogroup frequencies among populations (the Karitiana and Surui are 100% haplogroup D, the Pima are 100% haplogroup C, the Maya are 100% haplogroup A, and the Colombians are 50% haplogroup B and 50% haplogroup C; Additional file 3: Table S6), which are at least partly due to the small sample sizes but also in keeping with previous studies [70]. However, all NRY sequences from the Americas fall into haplogroup Q (with the exception of one Pima with a haplogroup G sequence that likely reflects recent European admixture), and overall NRY diversity is substantially reduced in the Americas, compared to mtDNA diversity (Table 1, Figure 2). While the small number of HGDP males from the Americas precludes any definitive statements, the apparently much greater mtDNA than NRY diversity in the Americas might indicate that fewer males than females were involved in the colonization of the Americas, and deserves further investigation.

We note some additional features pertaining to specific populations in the individual NRY haplogroup phylogenies provided in Figures S3 to S12 in Additional file 3, while the full mtDNA phylogeny for the HGDP samples is provided in Figure S13 in Additional file 3.

### Demographic history

**Figure 4:**
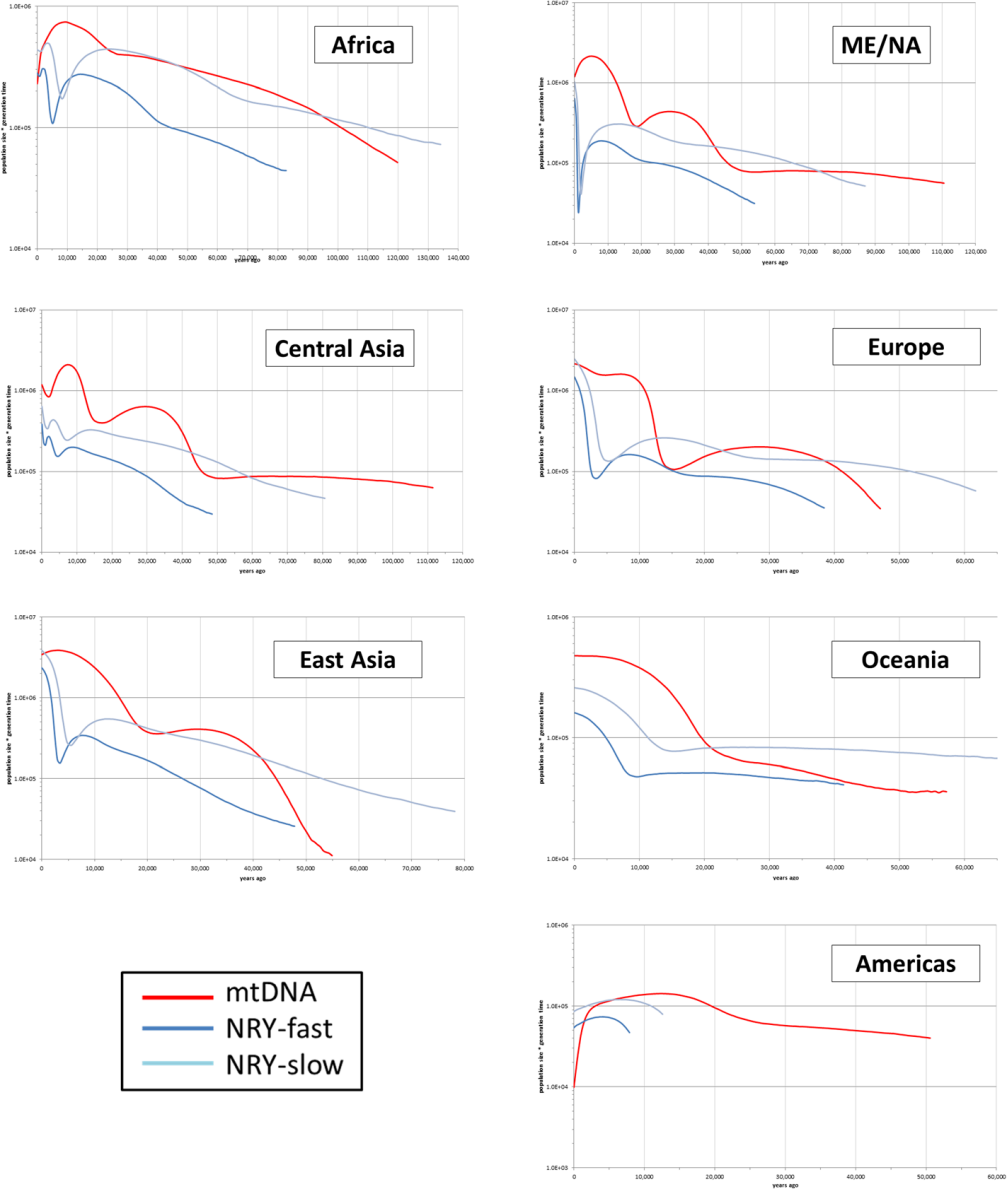
**Bayesian skyline plots of population size change through time for regional groups.** Two curves are shown for the NRY data, based on ‘fast’ and ‘slow’ mutation rate estimates.

Sequence-based analysis of NRY variation permits demographic analyses that cannot be carried out with ascertained SNP genotype data, and which can then be compared directly to similar analyses of the mtDNA sequences. In the following demographic analyses, only the sequence data were used, and not any of the haplogroup information. We first estimated the history of population size changes via Bayesian skyline plots (BSPs) for the NRY and mtDNA sequences for each region (Figure 4). These results should be interpreted cautiously, both because of the small sample sizes for some of the regions (in particular, America and Oceania), and because grouping populations with different histories can produce spurious signals of population growth [71]. Moreover, the uncertainty concerning the NRY mutation rate makes it more difficult to compare the timing of population size changes for the NRY *versus* mtDNA. Nevertheless, both the mtDNA and NRY BSPs indicate overall population growth in almost all groups, but for mtDNA there is a more pronounced signal of growth at around 15,000 to 20,000 years ago than there is for the NRY, and during much of the past it appears as if the effective size for females was larger than that for males (Figure 4).

To further investigate female and male demographic history, we used simulations and ABC to estimate the current and ancestral effective population size for females (N_f_) and males (N_m_) for Africa, Europe, East Asia, Central Asia, Oceania, and the Americas. We also estimated the ancestral N_f_ and N_m_ for the out-of-Africa migration. We first used the model in Figure 1 and the combined mtDNA and NRY sequences (using an average of the fast and slow mutation rates for the latter) to estimate the divergence times associated with this model (with the prior distributions for the divergence times given in Table 2). Table 2 also provides measures of the reliability of the resulting parameter estimation based on the pseudo-observed values: average R^2^ = 0.9, which exceeds the suggested threshold [72] of 10%; average coverage is 89% and factor 2 (proportion of estimated values for the statistics that are within 50% to 200% of the true value) is 90%; the average bias is 2% and relative mean square error (RMSE) is 9%. As these measures indicate satisfactory performance of the simulation [72], we retained the top 1,000 simulations (tolerance of 0.02%) for estimating the divergence times. In addition, the posterior distributions show a markedly improved fit to the summary statistics, compared to the prior distributions (Additional file 3: Table S7, Figure S14). The resulting estimates of divergence times for the model in Figure 1 are provided in Table 2, and are generally in good agreement with previous estimates for the divergence time among continental groups [45,73,74].

**Table 2:** Prior estimates of divergence time (all priors uniformly distributed) and the mean, mode, and 95% HPD (highest posterior density) intervals

**Parameter**	**Prior**	**Mean**	**Mode**	**95% HPD**	**R**^**2**^	**Bias**	**RMSE**	**Coverage**	**Factor 2**
T1	100,000-150,000	107,067	102,125	100,175-123,116	0.98	−0.01	0.07	95	1
T2	60,000-100,000	74,916	74,691	63,350-93,892	0.97	0.03	0.13	97	1
T3	60,000-100,000	63,210	61,152	60,200-67,718	0.98	0.01	0.05	100	1
T4	40,000-60,000	49,280	42,637	40,574-58,075	1	0.01	0.06	100	1
T5	20,000-40,000	36,700	38,394	30,475-39,581	0.91	0.03	0.09	92	1
T6	10,000-20,000	15,828	17,798	11,280-19,500	0.99	0.02	0.11	100	1

Simulations were based on combined mtDNA and NRY sequences and the model of population history shown in Figure 1. Also shown are various statistics related to 1,000 pseudo-observed parameter estimations: R^2^ is the proportion of the variance in the parameters explained by the summary statistics; Bias indicates whether the parameter tends to be over-estimated (positive bias) or under-estimated (negative bias); RMSE (root mean square error) is a distance between the true and estimated values of the parameter.

Coverage is the proportion of times the true value for the parameter lies within the 90% credible interval around the parameter estimate; and Factor 2 is the proportion of estimated values that are within 50% and 200% of the true value.

**Figure 5:**
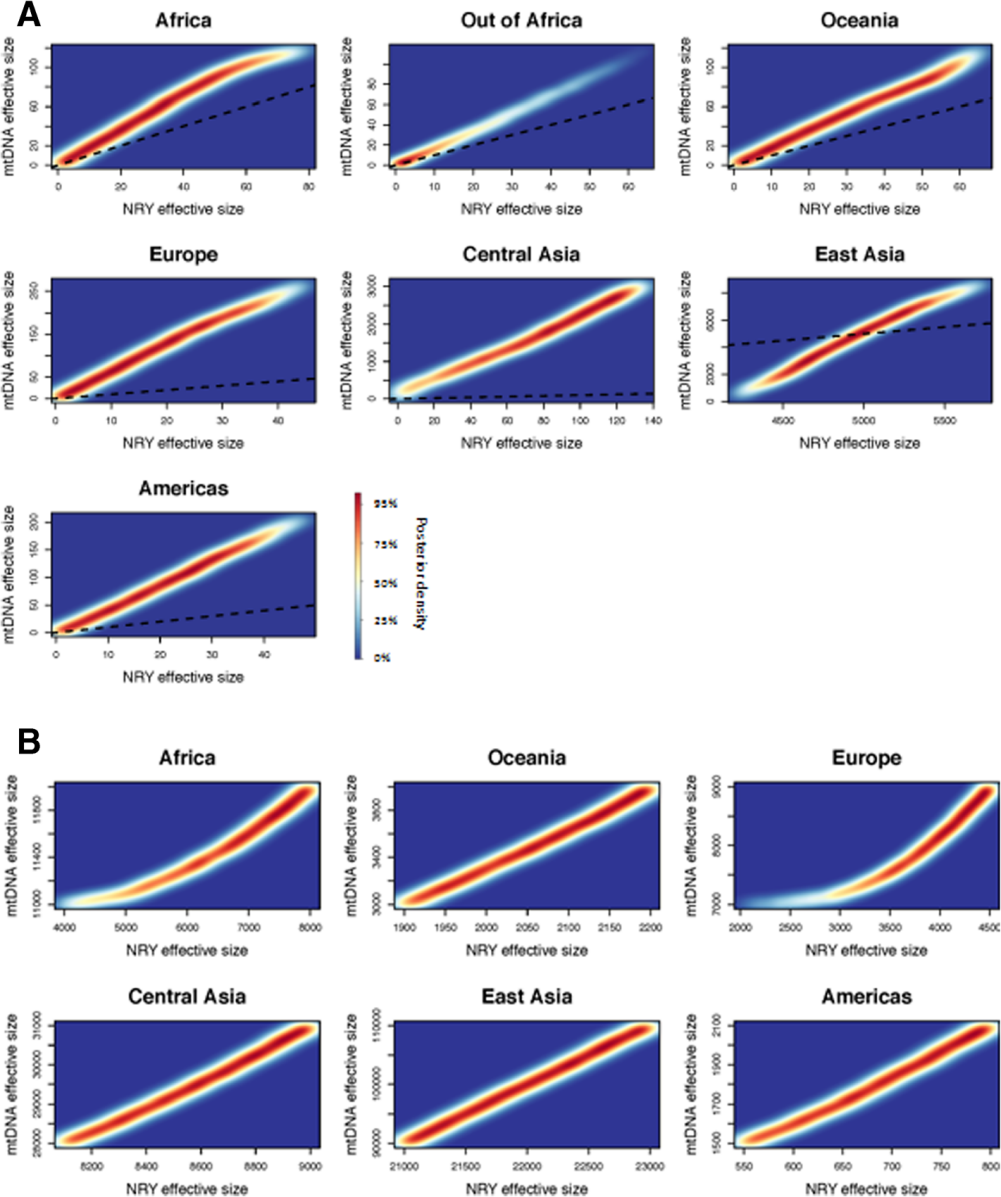
**Distribution of N**_**f **_**and N**_**m **_**values, based on simulations.** The density of the top 1% of the posterior values obtained from simulations of the mtDNA and NRY sequences are shown. **(A)** ancestral effective population sizes; **(B)** current effective population sizes. The dashed line in each plot follows a 1:1 ratio.

**Figure 6:**
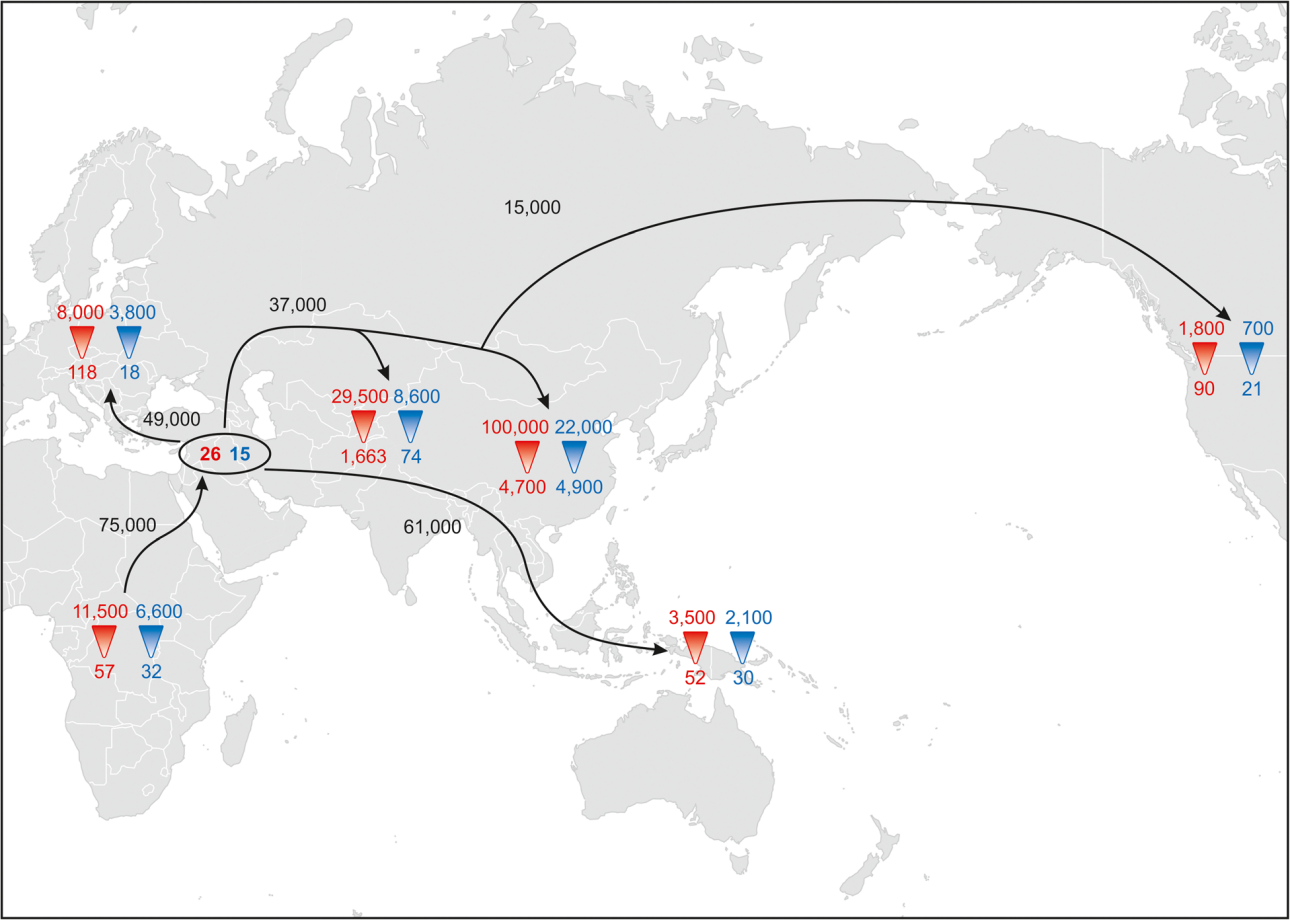
**Pictorial representation of the divergence time and female and male effective population size estimates, based on the simulation results.** Red numbers reflect N_f_ (with ancestral N_f_ at the point of the red triangle and current N_f_ at the base of the red triangle) and blue numbers correspondingly reflect ancestral and current N_m_. The numbers in the black oval indicate the founding effective sizes for the initial out-of-Africa migration, and dates on arrows indicate divergence times based on the model in Figure 1. Arrows are meant to indicate the schematic direction of migrations and should not be taken as indicating literal migration pathways, for example, the results indicate divergence of the ancestors of Oceanians 61,000 years ago, but not the route(s) people took to get to Oceania.

We next carried out separate simulations based on NRY and mtDNA sequences, respectively, and obtained ABC estimates of current and ancestral N_m_ and N_f_ for each regional group and for the out-of-Africa migration. Although the reliability measures indicate greater variance in the simulation results (Tables 3 and 4), the posterior distributions still show a markedly improved fit to the summary statistics (Additional file 3: Tables S8 and S9; Figures S15 and S16). The distribution of the estimated current and ancestral N_f_ and N_m_ are shown for each regional group in Figure 5, and a pictorial summary is provided in Figure 6. The simulation results suggest a small founding size in Africa of about 60 females and 30 males (all population sizes are effective population sizes); migration out of Africa about 75 kya associated with a bottleneck of around 25 females and 15 males; migrations from this non-African founding population to Oceania 61 kya, to Europe 49 kya, to Central and East Asia 37 kya, and from East Asia to the Americas about 15 kya. These divergence times are in reasonable agreement with those in the mtDNA and NRY phylogenies, given the wide confidence intervals on both (Table 2, Additional file 3: Table S4). There was concomitant population growth in all regions (with the most growth in East Asia); however, throughout history the mtDNA and NRY results indicate consistently larger effective population sizes for females than for males (except, possibly, in the ancestors of East Asians).

**Table 3:** **Current and ancestral estimates of male effective population size (N**_**m**_**) based on simulations of the HGDP NRY sequences**

	**Mean**	**Mode**	**95% HPD**	**R**^**2**^	**Bias**	**RMSE**	**Coverage**	**Factor 2**
*Current sizes*								
Africa	6,565	7,662	4,632-7,898	0.99	−0.01	0.11	100	1
Oceania	2,060	2,172	1,920-2,188	0.92	0	0.04	75	1
Europe	3,815	4,327	2,814-4,456	0.99	0.02	0.11	98	1
Central Asia	8,579	8,888	8,155-8,961	0.97	0	0.03	94	1
East Asia	22,009	22,630	21,113-22,901	0.96	0	0.03	81	1
Americas	685	746	566-789	0.95	0	0.11	79	1
*Ancestral sizes*								
Africa	32	48	2-75	0.69	2.97	2.62	81	0.63
Out-of-Africa	15	10	1-59	0.69	3.27	2.61	75	0.69
Oceania	30	12	3-62	0.67	1.91	2.19	88	0.56
Europe	18	17	1-42	0.70	2.77	2.43	83	0.62
Central Asia	74	122	10-129	0.78	1.18	1.09	89	0.78
East Asia	4,935	4,704	4,269-5,664	0.98	−0.02	0.07	89	1
Americas	21	28	2-45	0.58	2.41	2.39	80	0.64

The simulations assumed the model of population history in Figure 1 and the mean divergence time estimates in Table 2. Simulations were carried out with a uniform prior distribution on N_m_ of 1 to 100,000 for each regional group. The statistics for the pseudo-observed values (R^2^, Bias, RMSE, Coverage, and Factor 2) are as defined in the legend to Table 2.

**Table 4:** **Current and ancestral estimates of female effective population size (N**_**f**_**) based on simulations of the HGDP mtDNA sequences**

	**Mean**	**Mode**	**95% HPD**	**R**^**2**^	**Bias**	**RMSE**	**Coverage**	**Factor 2**
*Current sizes*								
Africa	11,505	11,841	11,052-11,951	0.93	−0.01	0.03	75	1
Oceania	3,509	3,936	3,053-3,952	0.98	−0.02	0.09	74	1
Europe	8,029	8,895	7,111-8,906	0.98	0.01	0.07	91	1
Central Asia	29,513	30,740	28,155-30,853	0.97	0	0.03	80	1
East Asia	100,111	108,787	91,032-109,030	0.97	0	0.06	71	1
Americas	1,802	2,030	1,531-2,070	0.97	0.04	0.10	78	1
*Ancestral sizes*								
Africa	57	10	5-113	0.67	1.96	1.88	82	1
Out-of-Africa	26	5	1-107	0.69	5.48	3.98	75	1
Oceania	52	13	4-112	0.65	2.09	2.21	90	1
Europe	118	23	10-253	0.88	3.09	2.77	73	1
Central Asia	1,663	2,863	372-2,956	0.91	0.19	0.41	97	1
East Asia	4,710	7,274	1,310-8,374	0.98	0.09	0.26	96	1
Americas	90	111	8-1,970	0.87	6.10	3.82	71	1

The simulations assumed the model of population history in Figure 1 and the mean divergence time estimates in Table 2. Simulations were carried out with a uniform prior distribution on N_f_ of 1 to 100,000 for each regional group. The statistics for the pseudo-observed values (R^2^, Bias, RMSE, Coverage, and Factor 2) are as defined in the legend to Table 2.

## Discussion

We report here the development and implementation of a capture-based array method to enrich Illumina sequencing libraries for NRY sequences. We then used this method to obtain approximately 500 kb of NRY sequence for 623 males from 51 populations of the CEPH-HGDP, and we also obtained complete mtDNA genome sequences from the same individuals. The molecular resolution (that is, number of SNPs) provided by the NRY and mtDNA sequences was roughly equivalent overall (2,228 NRY SNPs, *vs.* 2,163 mtDNA SNPs), allowing us to compare the maternal and paternal histories of human populations without the usual concerns about different methodologies (for example, mtDNA HV1 sequences *vs.* genotyping NRY SNPs and/or STRs) having an influence on the results. However, note that in other respects the molecular resolution still differs between the mtDNA and NRY sequences, for example, we obtained complete mtDNA genome sequences but only partial NRY sequences.

Our data provide new insights into the maternal *versus* paternal history of humans. First, a longstanding controversy has been whether or not genetic differences between human populations are bigger, on average, for the NRY than for mtDNA. The first comparative study of human mtDNA and NRY diversity found significantly bigger differences between populations for the NRY than for mtDNA [6], which was attributed to a higher female than male migration rate between populations due to patrilocality. A subsequent study found bigger differences between populations for the NRY than for mtDNA in patrilocal populations and the opposite pattern in matrilocal populations, [75] which was viewed as supporting an influence of residence pattern and associated male *versus* female migration rates on NRY *versus* mtDNA diversity. However, these studies used different methods to assay NRY *versus* mtDNA diversity; notably, a later study that used similar methods to assay NRY and mtDNA diversity (by comparing 6.7 kb of NRY sequence and 770 bp of mtDNA sequence in 389 individuals from 10 populations) concluded that genetic differences between populations were in fact similar for the NRY and mtDNA [7].

Our results, based on a more comprehensive sampling of worldwide human populations, indicate that genetic differences among human populations at the global scale are indeed bigger for the NRY than for mtDNA, although the differences are not as large as suggested by previous studies (between-population variance of 36% for the NRY *vs.* 25% for mtDNA in this study, compared to previous estimates of 65% for the NRY *vs.* 20% for mtDNA [6]). More importantly, our results indicate substantial differences among regional groups in the between-group variance for the NRY *versus* mtDNA (Figure 2) as well as in overall levels of NRY *versus* mtDNA diversity (Figure 2, Table 1). Thus, focusing on global patterns of variation misses this important regional variation, which (as discussed in more detail above in the Results) likely reflects differences in the paternal *versus* maternal demographic history of specific human populations (for example, the large impact of the Bantu expansion on African NRY diversity [59,60], and of the Austronesian expansion on Oceanic mtDNA diversity [63,65]).

Another question of interest is the extent to which the genetic contributions of males *versus* females have been the same or differed (as measured by their respective effective population sizes, N_m_ and N_f_, respectively). Previous studies of N_m_ and N_f_ have largely relied on comparisons of X chromosome vs. autosomal variation, and have come to varying conclusions concerning the historical N_f_/N_m_ ratio, for example, finding that this ratio suggests a large excess of N_f_ to N_m_ [8], a moderate excess of N_f_ to N_m_ [76], or even a decreased N_f_ relative to N_m_ [9]. These differences variously reflect methodological differences, difficulties in accounting for differences in male *versus* female mutation rates, and/or the potentially greater effect of selection on the X chromosome than on the autosomes [10,11]. Comparison of mtDNA *versus* NRY variation offers a more direct assessment of N_f_/N_m_ that is free of some of the issues concerning X:autosome comparisons (albeit not all, as discussed below), but requires unbiased estimates of NRY variation, which until our study were only available from either whole genome sequencing studies [5,14-16] or more limited targeted studies of NRY sequence variation [7,77]. Our results indicate a consistent strong excess of N_f _*versus* N_m_ starting even before the out-of-Africa migration that has been carried through almost all subsequent migrations. East Asia may be an exception, and indeed our estimates of N_f_ and N_m_ are substantially larger than previous estimates of N_e_ in east Asians based on autosomal diversity [78,79]. However, these previous studies were based solely on data from Han Chinese and Japanese, whereas the HGDP includes a much more diverse sampling of east Asian populations, which may account for the higher effective population size estimates for the HGDP. The excess of N_f_*versus* N_m_ become even more pronounced in recent times due to higher rates of growth in N_f_ than in N_m_ (Figures 4, 5, and 6); these results are in line with previous studies of smaller datasets that used different methods [4,80]. These results suggest, in turn, that sex-specific processes that reduce N_m_, such as polygyny and/or sex-specific migration [2], have characterized humans over most of our prehistory.

However, there are several reasons why this conclusion should be viewed as tentative. First, the sample sizes of some of the regional groups in the HGDP are quite low, precluding confident estimates of effective population sizes. Moreover, there are some surprising features of our results, such as the much larger effective size estimates for East and Central Asians than for Europeans. Whether these features are truly indicative of these regions, or rather specific to the particular populations sampled in the HGDP, will require further studies to elucidate. Nonetheless, given that the HGDP overall is a much more comprehensive sampling of worldwide genetic diversity than in previous studies that estimated effective population sizes for various human populations, it perhaps is not surprising that we obtain different results.

Second, while focusing on NRY *versus* mtDNA variation avoids some of the drawbacks of comparing X *versus* autosomal DNA variation in estimating N_f_ and N_m_, the uncertainty associated with the resulting estimates is significantly larger for NRY:mtDNA than for X:autosome comparisons. This is because the X:autosome comparisons are averaged across many independent loci, whereas the NRY and mtDNA are each just a single independent locus.

Third, the model used in the simulations is obviously a very simplified version of reality, and indeed there are some clear differences between the observed values for some summary statistics and the posteriors (for example, the Φ_ST_ values in Figures S14 to S16 in Additional file 3). In particular, to reduce the computational complexity we did not consider migration between regional groups (after the initial colonization events) in the simulations to estimate N_f_ and N_m_. There is some justification for doing so, as in general migration within the regional groups has been more important than migration between regional groups, as evidenced by genetic structure analyses [43,47,81] and by attempts to estimate migration rates directly from genetic data [80]. Moreover, no mtDNA sequences are shared between regional groups, and only one NRY sequence is shared between regional groups, suggesting very limited recent migration between regional groups. Furthermore, by not including migration we are overestimating the ancestral N_f_ and N_m_ (because some of the diversity reflects later migration rather than genetic diversity that was present in the ancestral population). Thus, the effect of such migration would be even smaller estimates of N_f_ and N_m_ than those we obtained. Still, in future analyses migration and other complexities should be considered.

Fourth, we have here interpreted differences in levels of NRY *versus* mtDNA diversity and divergence as reflecting neutral, demographic history. However, a recent study has shown that background selection on the Y chromosome is probably also influencing levels of NRY diversity in human populations [50]. The substantial regional variation that we see in comparisons of mtDNA *versus* NRY diversity does suggest that there are regional differences in the demographic history of males and females, as it seems unlikely that levels of background selection would vary so drastically across human populations. Moreover, recently-described regional variation in ratios of X:autosomal diversity also point to sex-biased demographic processes [12]. Still, the overall differences we find in N_m_*versus* N_f_ may be influenced by background selection, and hence may not be as large as inferred by the simulations (for example, Figure 6). More detailed investigations are warranted into the relative importance of background selection *versus* purely demographic processes in influencing regional variation in N_m_ vs. N_f_.

## Conclusions

We have developed a rapid and cost-effective means of obtaining unbiased, high-resolution NRY sequence information. Comparative analysis of NRY and mtDNA sequences from a large sample of individuals and populations from the HGDP provides new insights into the comparative demographic history of males and females. In particular, we find on average larger genetic differences between populations for the NRY than for mtDNA (albeit with substantial regional variation), and that the effective population size of females has been larger than that of males throughout human history. We anticipate that using this approach to investigate additional populations should provide a rich source of new information about the genetic history of our species.

## Abbreviations

ABC: Approximate Bayesian computation; AMOVA: Analysis of Molecular Variance; APE: Analyses of Phylogenetics and Evolution; bam: Binary alignment map; BEAST: Bayesian Evolutionary Analysis Sampling Trees; bp: Base pairs; BSP: Bayesian Skyline Plot; BWA: Burrows-Wheeler Aligner; ESS: Effective sample size; GATK: Genome Analysis Toolkit; GTR: Generalized time-reversible; HGDP: Human Genetic Diversity Panel; HKY: Hasegawa-Kishino-Yano; IBIS: Improved Base Identification System; kb: Kilobases; kya: Thousand years ago; MCMC: Markov Chain Monte Carlo; ME/NA: Middle East and North Africa; MIA: Mapping Iterative Assembler; mtDNA: Mitochondrial DNA; mpd: Mean number of pairwise differences; MUSCLE: Multiple Sequence Comparison by Log Expectation; N_f_: Female effective population size; N_group_: Group sample size; N_m_: Male effective population size; ng: Nanograms; nmol: Nanomoles; NRY: Non-recombining Y chromosome; nt: Nucleotides; PHYLIP: Phylogeny Inference Package; qPCR: Quantitative polymerase chain reaction; RMSE: Relative mean square error; SNP: Single-nucleotide polymorphism; STR: Short tandem repeat; TrN: Tamura-Nei; UCSC: University of California Santa Cruz; VCF: Variant Call Format.

## Competing interests

The authors declare that they have no competing interests.

## Authors’ contributions

MS and SL conceived and designed the study. SL, AB, and RS performed the experiments. SL, HX, AK, ML, and GR analyzed the data. MS wrote the manuscript with input from all authors. All authors read and approved the final manuscript.

## Supplementary Material

Additional file 1: Table S1 Population, regional group affiliation, coverage, and haplogroup assignments for the HGDP samples analyzed here. Click here for file

Additional file 2: Table S2 NRY regions chosen for inclusion on the capture array. Click here for file

Additional file 3 **Supplement.** Additional supplementary tables and figures. Click here for file
