# Supplementary material for: Human paternal and maternal demographic histories: insights from high-resolution Y chromosome and mtDNA sequences
Source: Investig Genet. 2014 Sep 24;5:13. doi: 10.1186/2041-2223-5-13 (PMC4174254; doi:10.1186/2041-2223-5-13)
Supplement: Additional file 3 — Supplement. Additional supplementary tables and figures. [file 2041-2223-5-13-S3.pdf]

Supplementary Information

Human paternal and maternal demographic histories: insights from high-resolution Y chromosome and mtDNA sequences

Sebastian Lippold, Hongyang Xu, Albert Ko, Anne Butthof, Mingkun Li,

Gabriel Renaud, Roland Schröder, Mark Stoneking

This supplement contains Supplementary Figures 1-16, Supplementary Tables 3-8 (supplementary tables 1-2 are provided as separate data files), and associated References.

Figure S1. Average coverage for the NRY (top) and mtDNA (bottom) sequences.

NRY

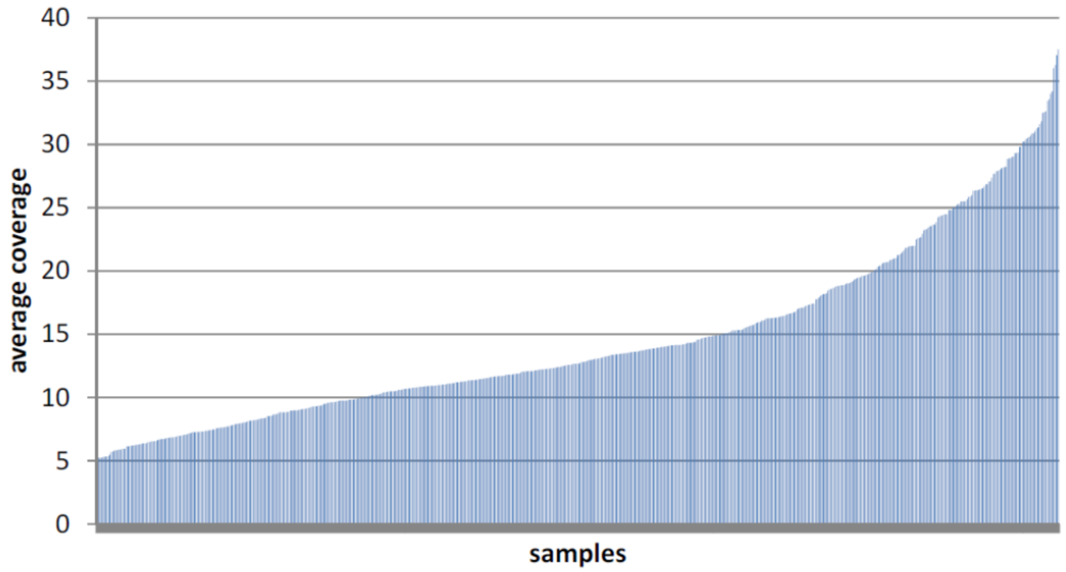

mtDNA

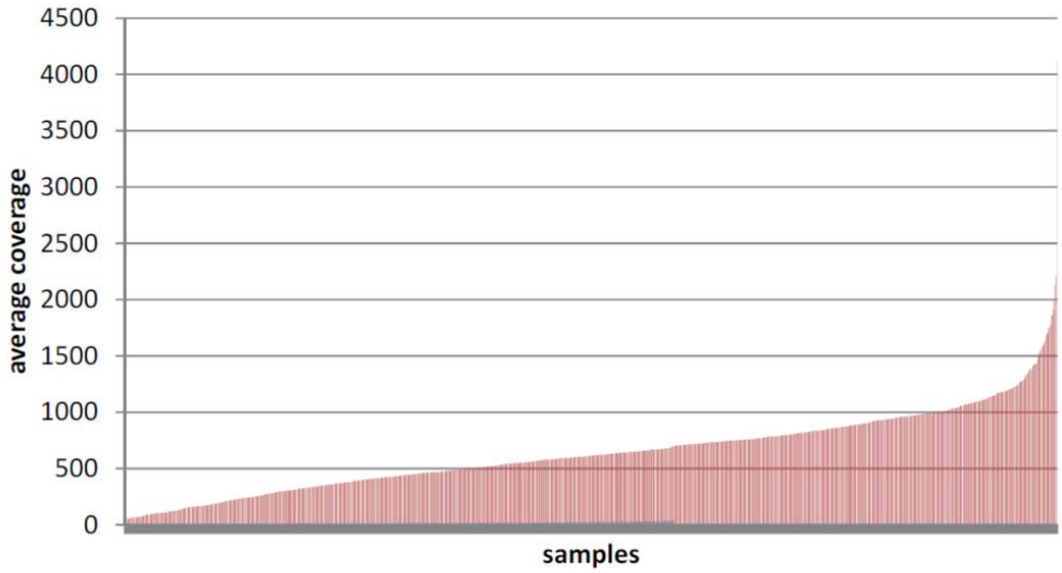

Figure S2. Comparison of diversity statistics based on mtDNA genome sequences from the 623 males vs. all 952 individuals from the HGDP. (**a**) nucleotide diversity, (**b**) mean number of pairwise differences.

a

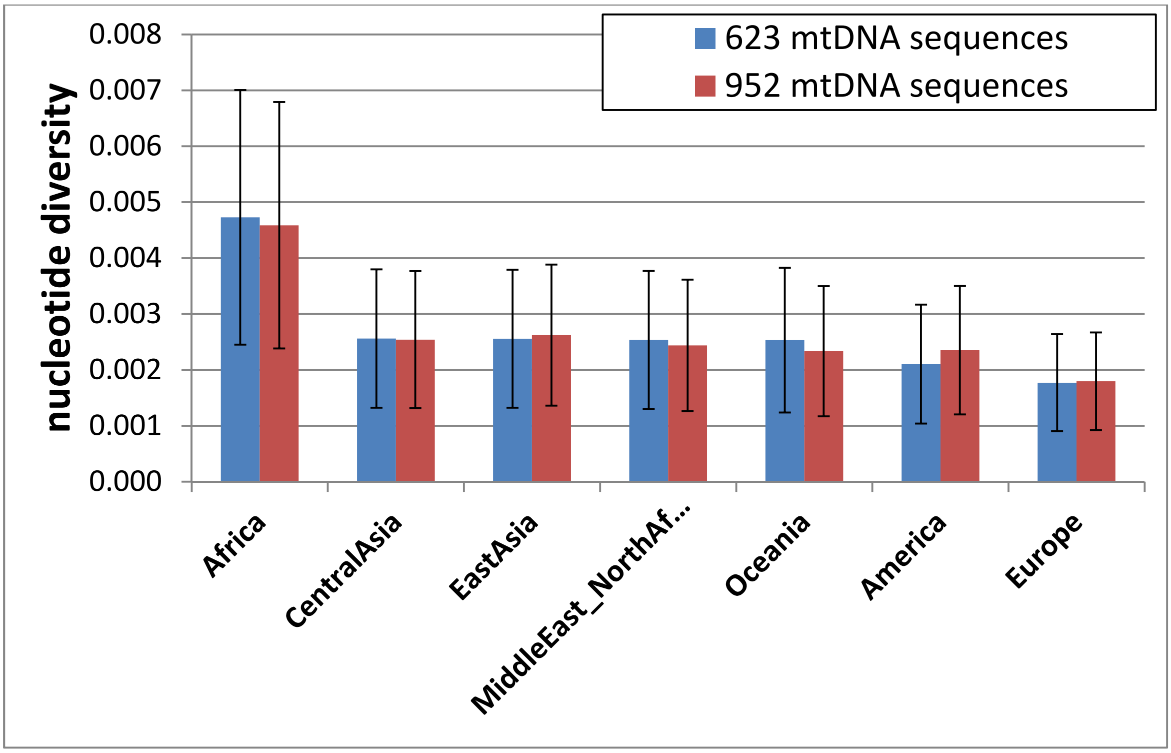

b

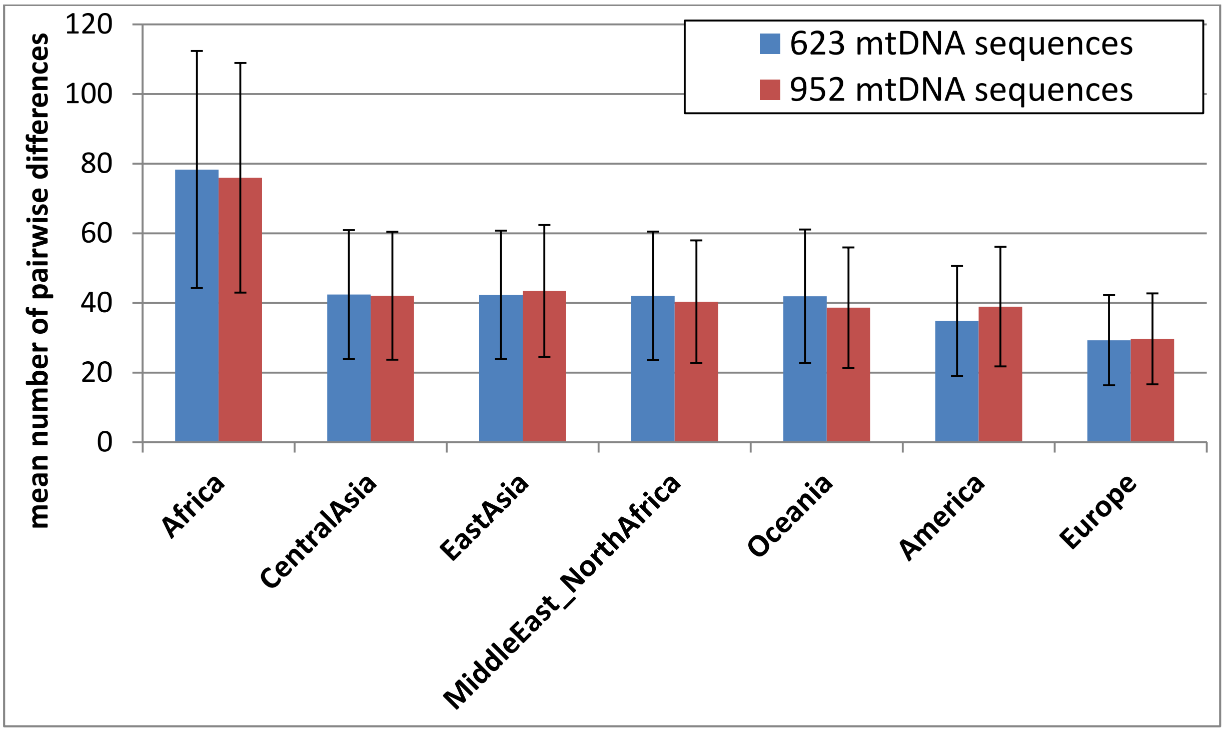

Figure S3. **Bayesian tree for sequences from NRY haplogroups A and B.** In this and subsequent trees, all dates are based on the "fast" rate, and tips are labeled with the HGDP ID number followed by haplogroup as determined previously by SNP typing [1, 2]. The only sublineage information in this tree is for haplogroup B2b4, which is monophyletic but does contain one haplogroup B sequence that was not genotyped as haplogroup B2b4.

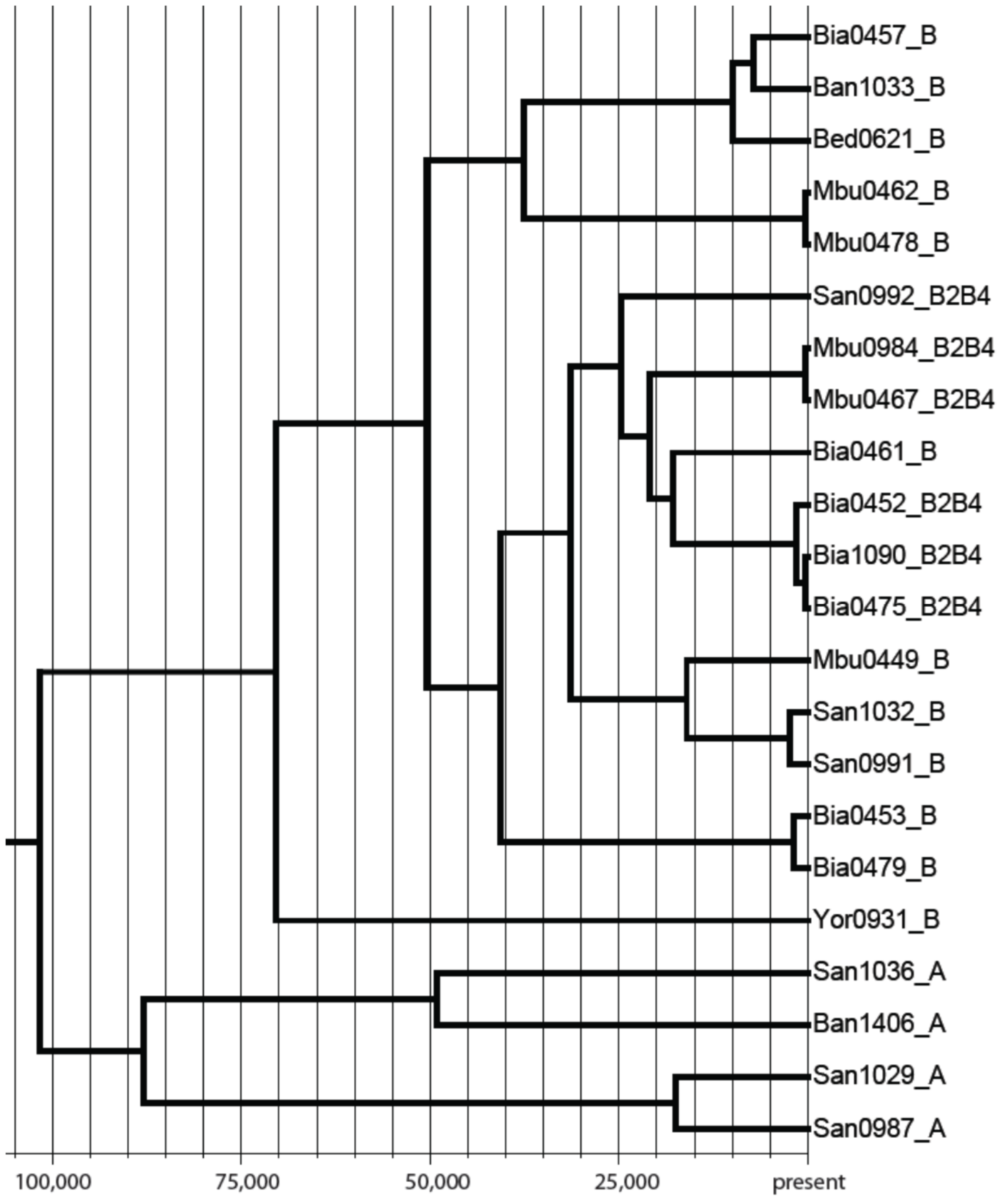

Figure S4. **Bayesian tree for NRY haplogroup C sequences.** The estimated age for this haplogroup is about 45 kya, in good agreement with previous estimates [3-5]. Although several sublineages of haplogroup C are known [6], and there are several clades evident in the tree, the HGDP samples have not been typed for haplogroup C sublineages. The arrow indicates a clade of 6 sequences from the Hazara that dates to about 2 kya. This clade is nested within a clade of sequences from populations from inner Mongolia, suggesting a migration from this region to the Hazara at around this time.

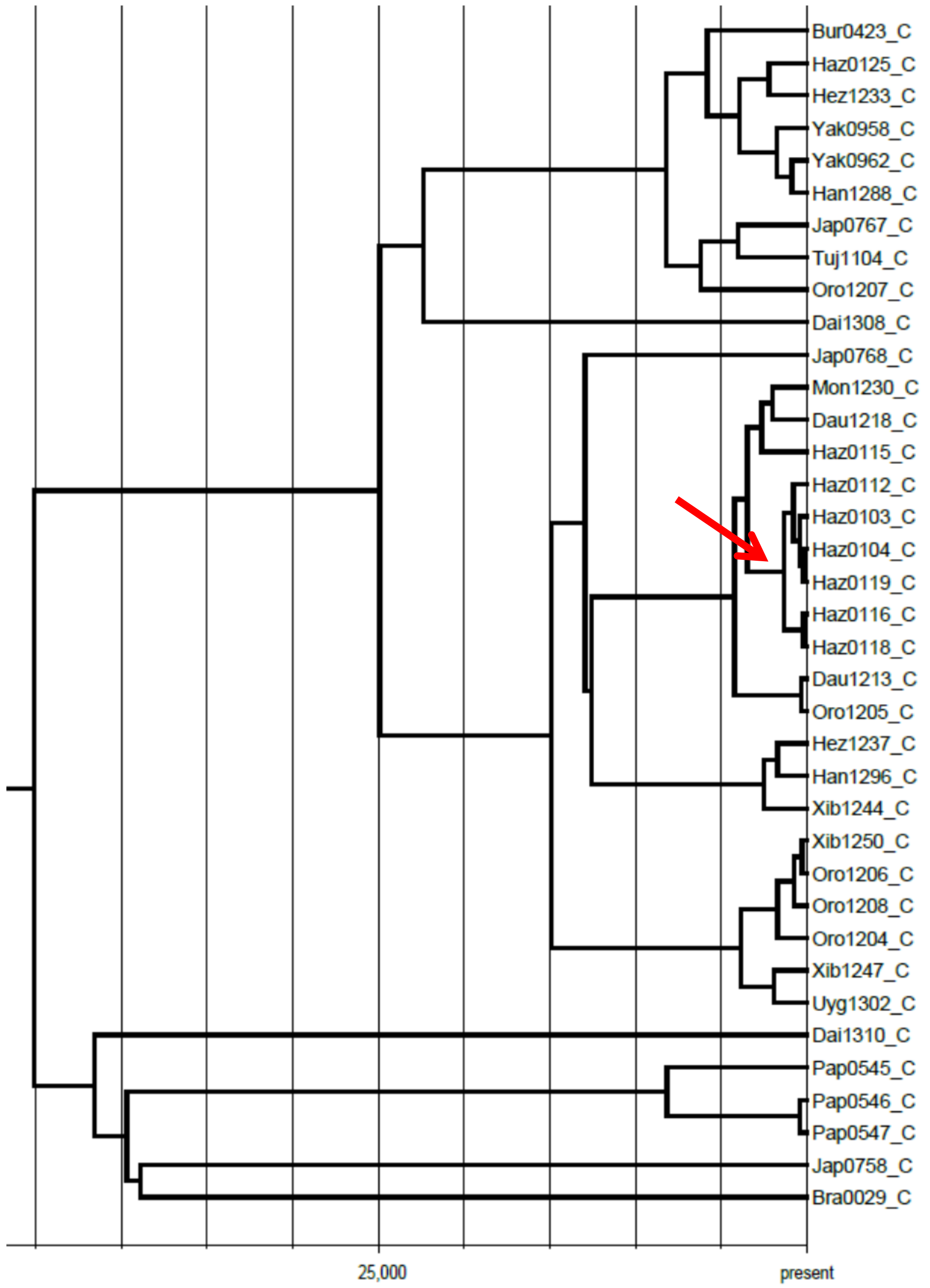

Figure S5. Bayesian tree for NRY haplogroup D sequences. The estimated age of this haplogroup is about 34 kya, more recent than previous estimates [3, 7], which could reflect incomplete sampling of haplogroup D diversity in the HGDP populations. The three major sublineages of haplogroup D [6] are all resolved in the tree; moreover, the sequences suggest that D1 and D3 are more closely related, whereas the current NRY tree has D1, D2, and D3 as a trichotomy [6]. The Japanese-specific clade of D2 sequences has an estimated age of about 17 kya, in good agreement with previous dates for the expansion of haplogroup D in Japan [8].

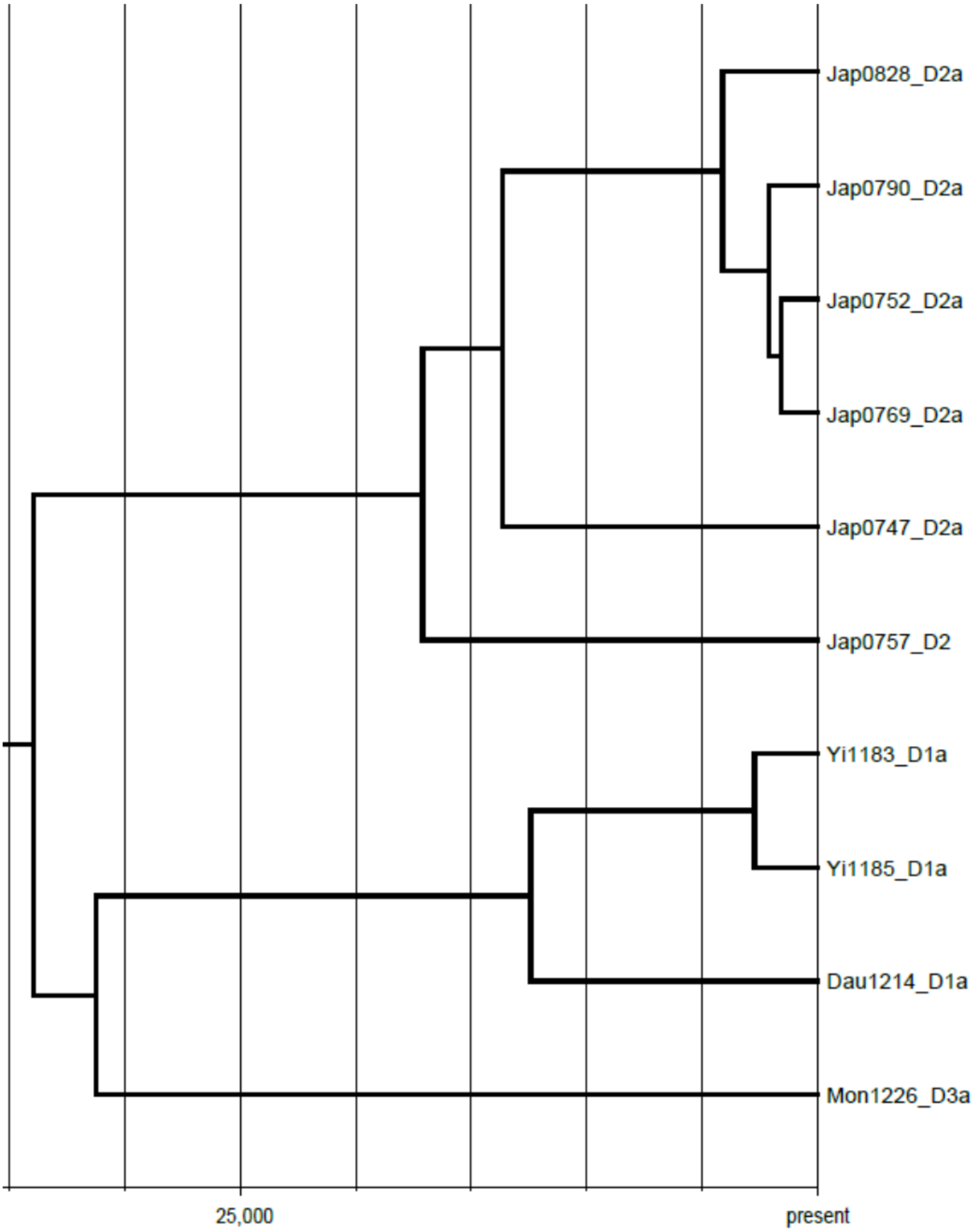

Figure S6. Bayesian tree for NRY haplogroup E sequences. There are four major clades in the tree, corresponding to haplogroups E2, E1a, E1b1a and sublineages, and E1b1b1 and sublineages. Some sublineages are monophyletic (e.g., E1b1b1b) while others are not (e.g., E1b1a7a). The red arrow points to a clade that contains most of the non-African sequences, while a blue arrow indicates the subclade that contains all of the European sequences.

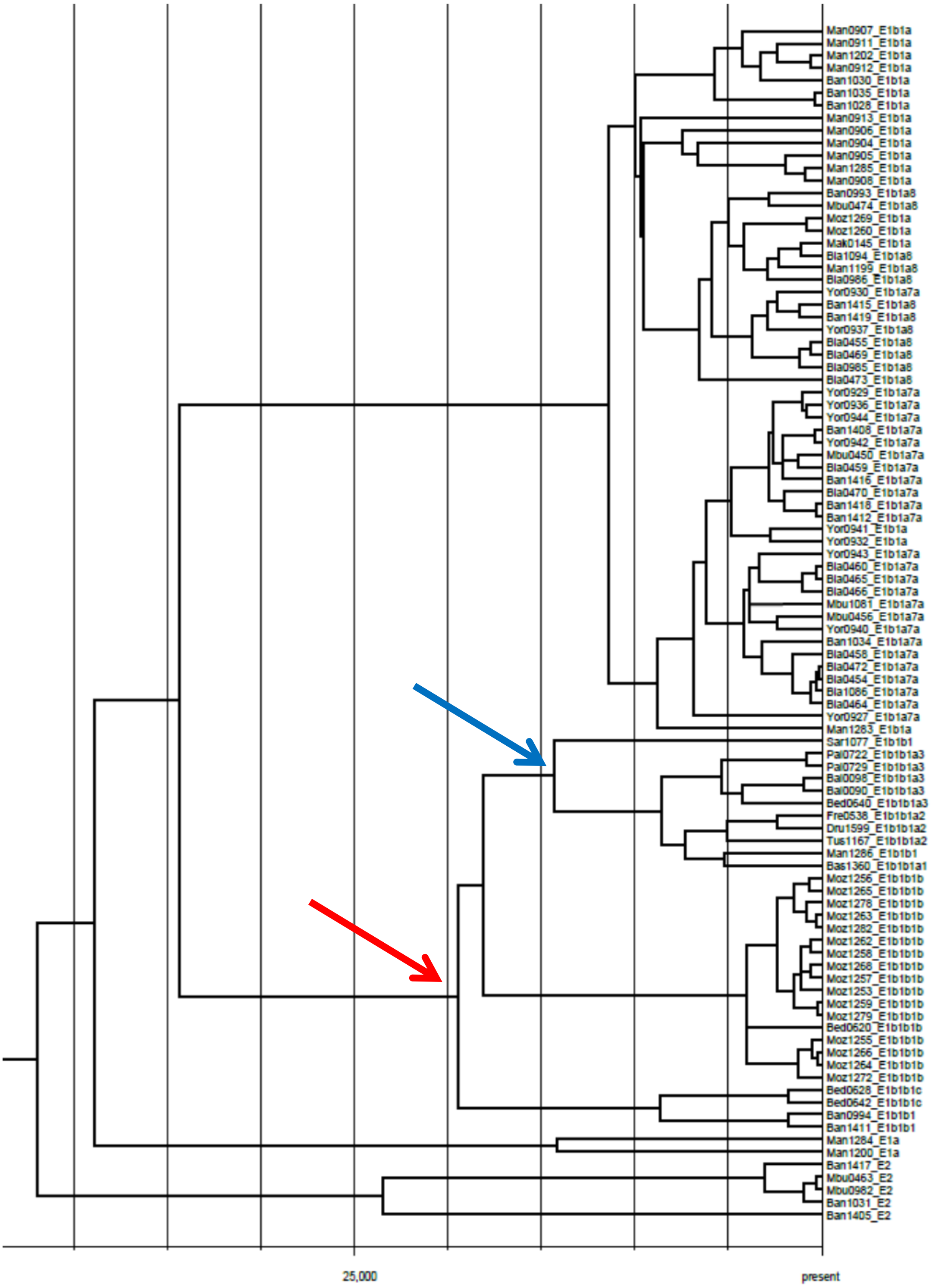

Figure S7. Bayesian tree for sequences from NRY haplogroups F and G. Haplogroup F in the HGDP is found only in the Lahu population, as haplogroup F2. Haplogroup G has an estimated age of about 28 kya in our data, older than previous estimates of 9.5-17 kya based on STR data [9, 10], but more in line with recent sequence-based studies [11, 12]. The origin of haplogroup G has been placed in the Caucasus or the Middle East [6], whereas the deepest divergences in our haplogroup G tree are in central Asia. There is a clade of 11 haplogroup G sequences that is specific to Palestinians (arrow) and has an age of about 2 kya, but diverged from other haplogroup G sequences (mostly from Europe, along with one native American Pima sequence that probably reflects recent European admixture) about 10 kya. The deep divergence and recent age for this clade suggests a possible bottleneck or founder event in the history of Palestinians.

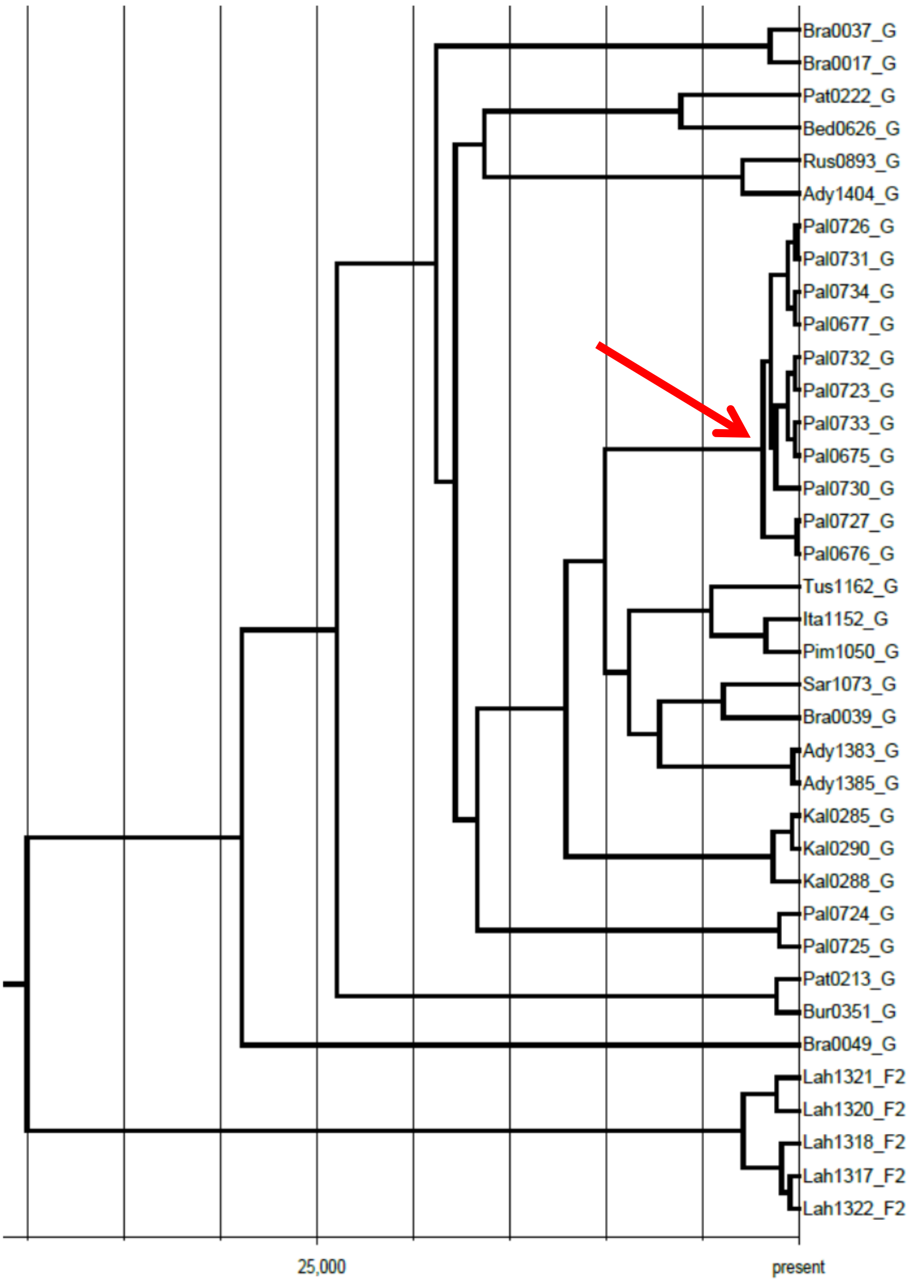

Figure S8. Bayesian tree for sequences from NRY haplogroups H, K, M, and L. Also included is one haplogroup F sequence that was placed with haplogroup H sequences rather than other haplogroup F sequences, but the support for this grouping is very low. Haplogroups H, H1, and L are monophyletic, but some sequences from haplogroup K form a monophyletic clade (red arrow) while others are interspersed among haplogroup M1 sequences (blue arrow). Haplogroup H has an estimated age of about 31 kya in our data, while haplogroup H1 has an age of 12.5 kya, in good agreement with previous estimates [13]. Our estimated age for haplogroup L is about 15 kya, slightly older than a previous estimate of 10 kya based on STR variation [13]. Haplogroups H and L are found mostly in central and east Asians in the HGDP. Haplogroups K and M1 are found exclusively in Oceania (with the exception of one Xibo with a haplogroup K sequence), and the clade of exclusively haplogroup K sequences has an estimated age of about 35 kya, in good agreement with previous estimates for Oceanic haplogroup K sequences [5, 6]. The clade with both M1 and K sequences (blue arrow) has a much younger age, only about 11 kya, suggesting a different history for these sequences than for the more divergent clade of exclusively haplogroup K sequences (red arrow).

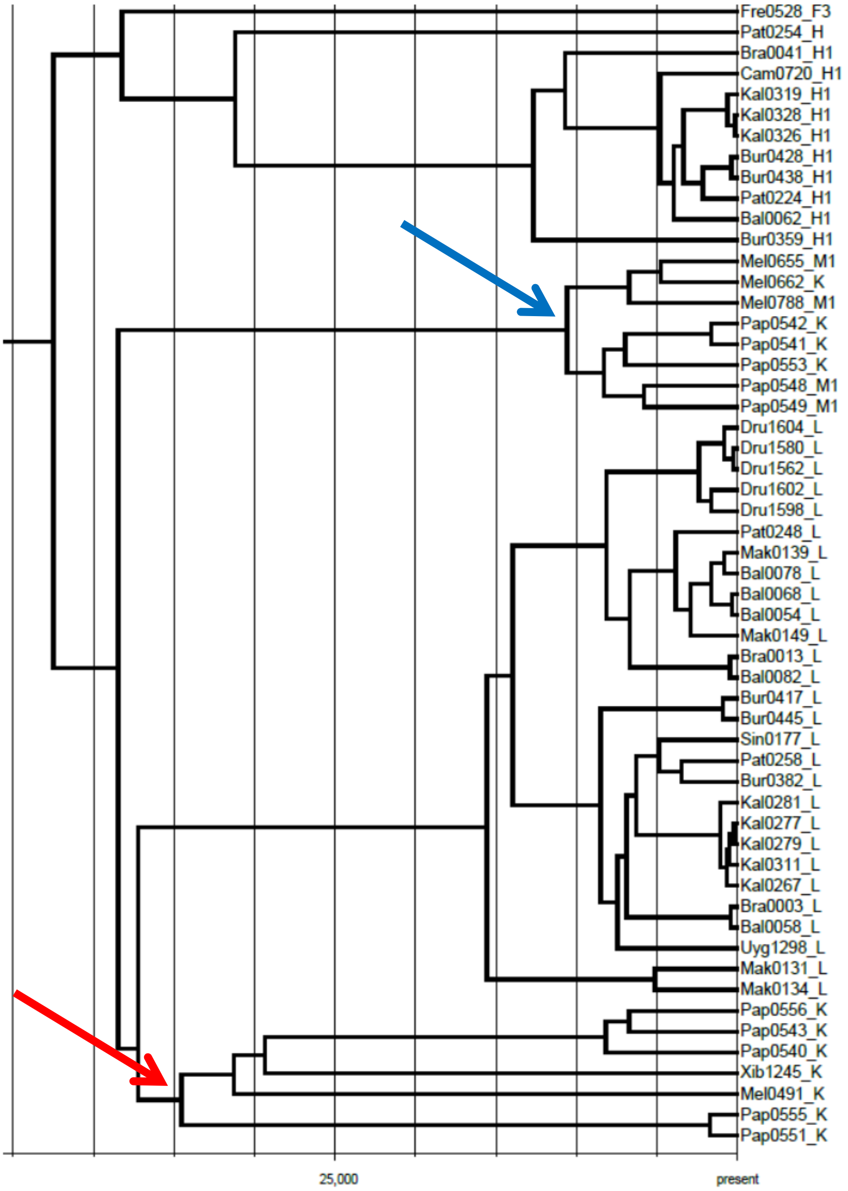

Figure S9. Bayesian tree for sequences from NRY haplogroups I and J. Sequences from both haplogroups form monophyletic clades, as does subhaplogroup J2; haplogroup I has an estimated age of about 17.5 kya, while haplogroup J has an estimated age of about 23 kya. Haplogroup I is exclusively European in the HGDP, with a clade of 10 Sardinian sequences (red arrow) with an estimated age of nearly 5 kya. Haplogroup J HGDP sequences come from Asia, the Middle East, and Europe; there is a clade in this haplogroup of 14 Bedouin haplogroup J sequences (blue arrow) with an age of about 4 kya.

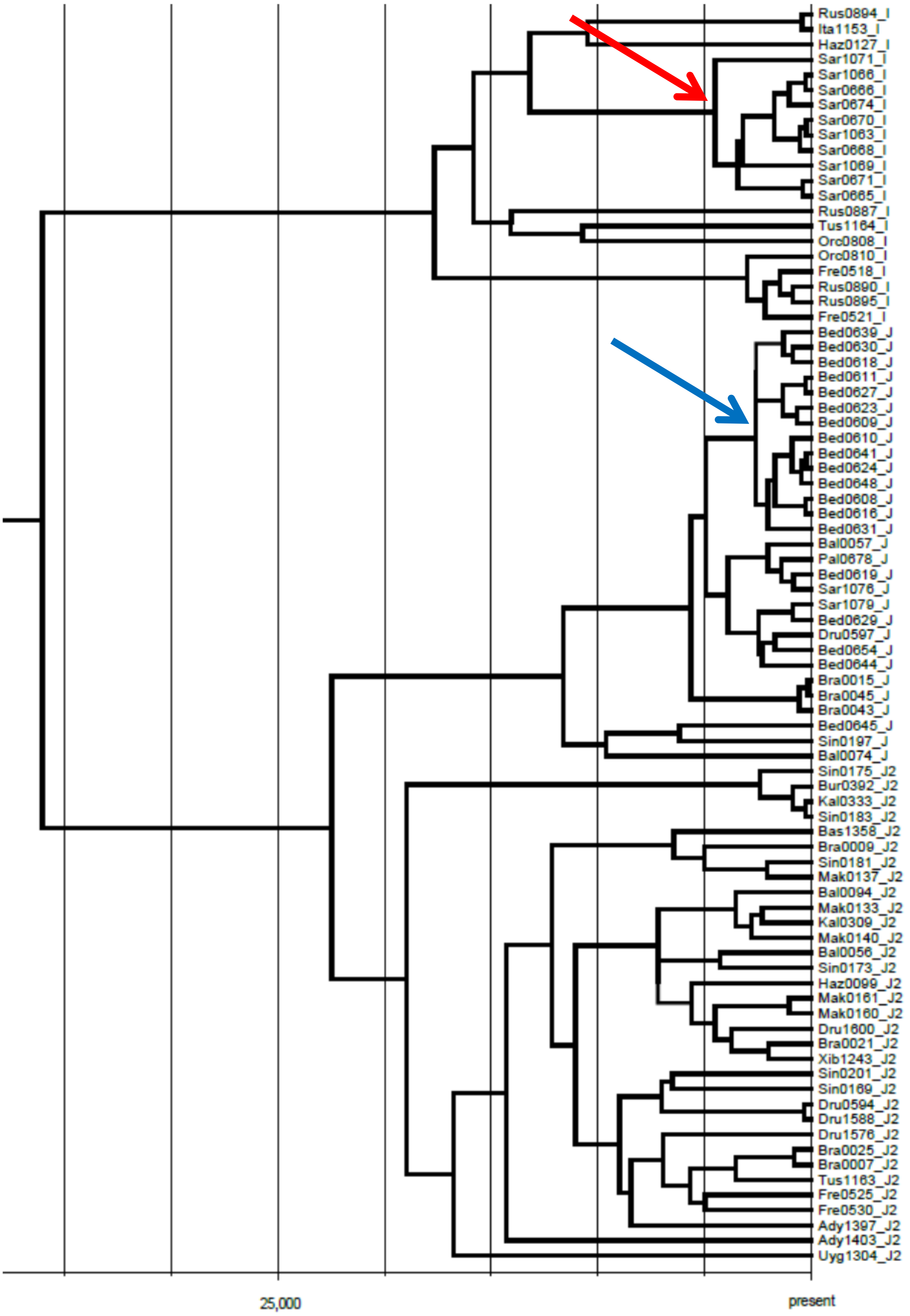

Figure S10. Bayesian tree for sequences from NRY haplogroups NO, N and O. Haplogroup NO is considered the ancestor of haplogroups N and O [6, 12], although in the HGDP tree haplogroup NO sequences are related to haplogroup N sequences (represented in the HGDP by only haplogroup N1c). Haplogroup NO sequences in the HGDP are all from east Asia. Haplogroup N1c has an estimated age of just over 10 kya in our data, in good agreement with previous estimates [14]. There is a clade of 16 Yakut N1c sequences with an age of nearly 6 kya (red arrow). High frequencies of haplogroup N1c sequences have been reported previously in Yakuts, but the estimated age in the present study is much older than previous estimates of less than 1 kya for N1c in Yakuts [15]; more sampling of Siberian groups are needed to determine if the clade of N1c sequences in our study are really specific to Yakuts. Haplogroup O has an estimated age of 27.5 kya based on the HGDP sequences, in good agreement with previous estimates [5]. The major subhaplogroups of haplogroup O in the HGDP data include O3, with an estimated age of 21 kya, and haplogroup O3a3c, with an estimated age of 13 kya; haplogroup O sequences are almost exclusively from east Asia.

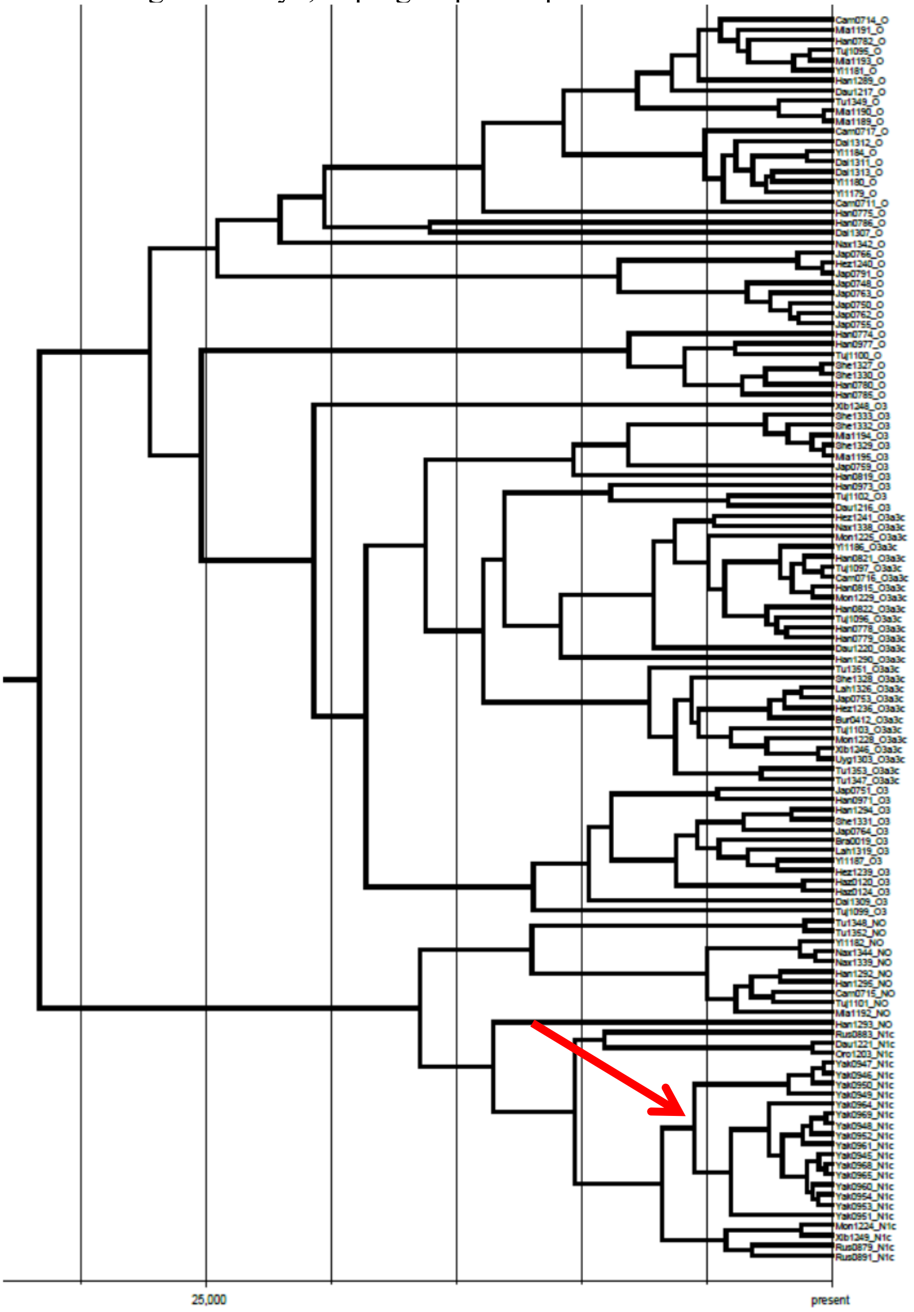

Figure S11. Bayesian tree for sequences from NRY haplogroup Q. Haplogroup Q has an estimated age of 28 kya based on the HGDP sequences, older than previous estimates [16]. There are three Central Asian haplogroup Q* sequences in the HGDP, while within haplogroup Q1a there is a clade (arrow) that contains almost exclusively sequences from the Americas, along with one Mongolian sequence. The age of this clade is about 12.5 kya, in good agreement with current evidence for the initial colonization of the Americas [17].

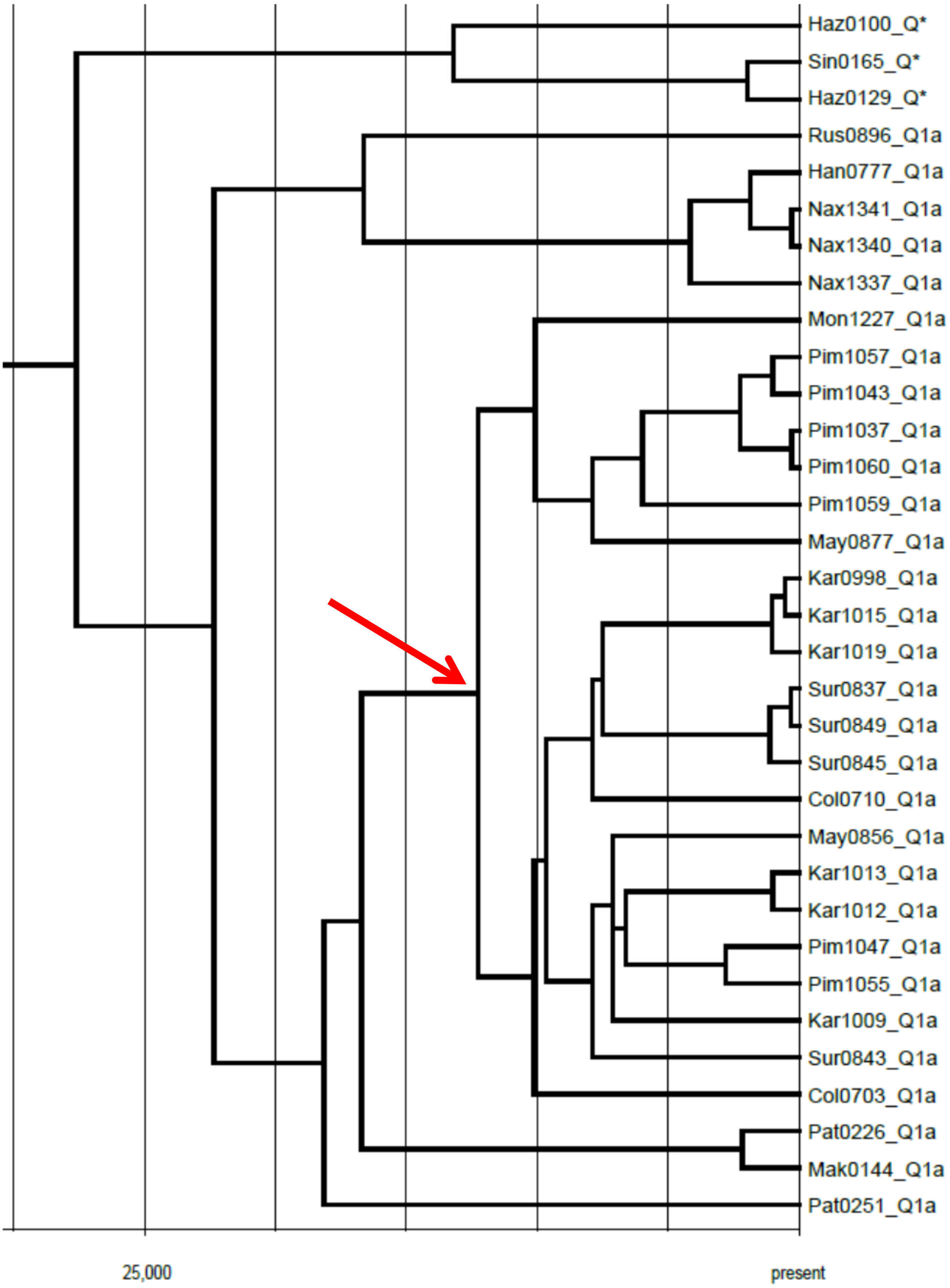

Figure S12. Bayesian tree for sequences from NRY haplogroup R. This haplogroup has an estimated age of 21 kya based on the HGDP sequences, slightly younger than previous estimates of about 27 kya [6]. Whereas in the SNP-based tree of NRY haplogroups R* is ancestral to other R haplogroups, in the HGDP tree the R* sequences diverged from R2 sequences about 11 kya, while R1a1 sequences diverged from R1b1, R1b1b1, and R1b1b2 sequences about 18 kya. R* and R2 sequences are almost exclusively from central Asia, while R1a1 and R1b1 sequences are more widespread and include central Asia, the Middle East, and Europe (mostly Adygei and Russians from eastern Europe). The R1b1b1 sequences are only from the Hazara, and diverged from R1b1b2 sequences (which are almost exclusively European) about 11 kya. The diversity within R1b1b2 dates to about 8 kya, so the age and geographic distribution of subhaplogroup R1b1b2 suggest a possible spread to Europe and expansion during the Neolithic [11, 18].

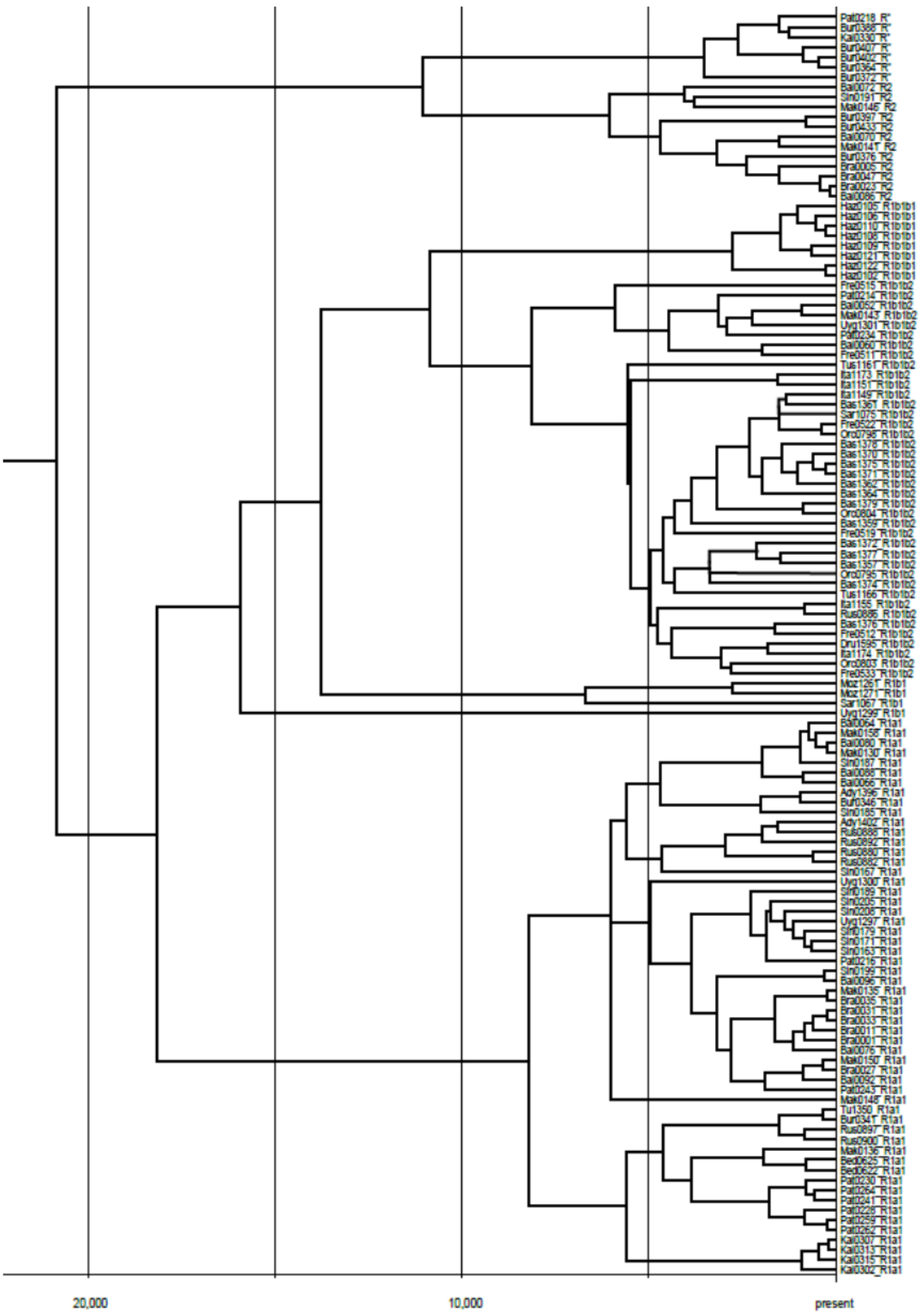

Figure S13. Bayesian tree of mtDNA sequences. As the human mtDNA phylogeny based on complete mtDNA sequences has been thoroughly documented [19], we do not comment on the details.

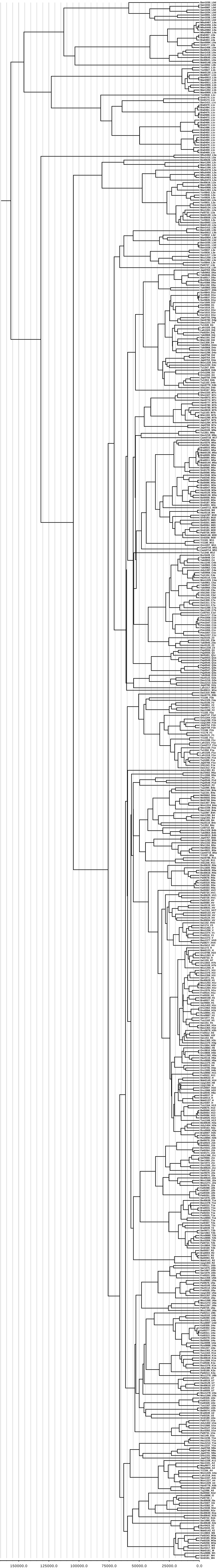

150000.0
125000.0
100000.0
75000.0
50000.0
25000.0
0.0

Figure S14. **Prior and posterior distributions of the summary statistics used in the simulations to estimate population divergence times.** Simulations were based on combined mtDNA and NRY sequences; blue indicates the prior distribution, red the posterior distribution, and the vertical black line is the observed value. Regional groups are numbered as follows: 1-Africa; 2-Oceania; 3-Europe; 4-Central Asia; 5-East Asia; 6-Americas. S is the number of polymorphic sites, D is Tajima's D value; Pi is the mean number of pairwise differences, FST is the Φ_{ST} value between each pair of groups, and Va, Vb, and Vc are the among group, among populations within groups, and within population variance components of an AMOVA with two groups (African populations and non-African populations).

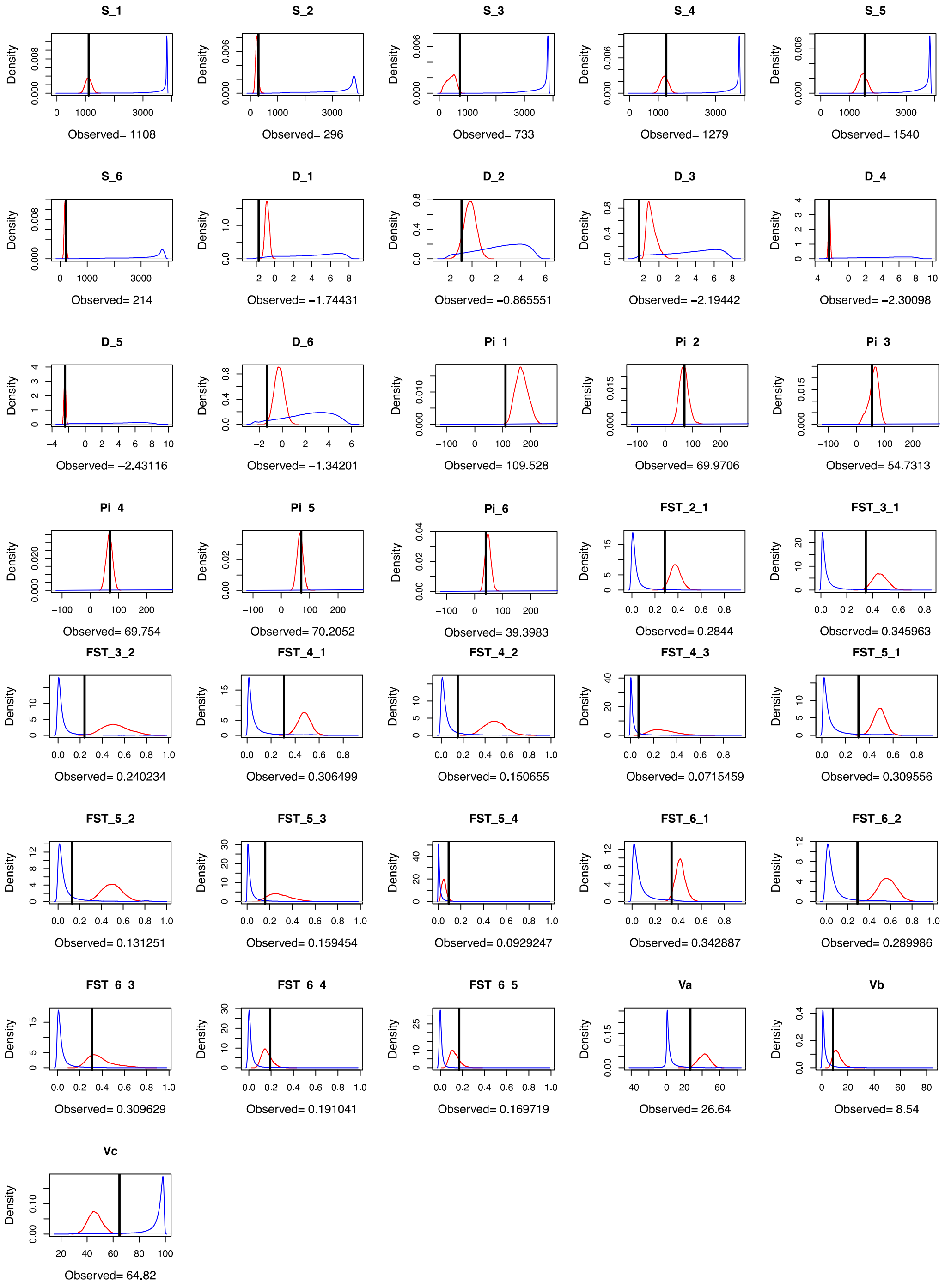

Figure S15. **Prior and posterior distributions of the summary statistics used in the simulations to estimate N_m for each regional group.** See the legend to **Figure S14** for further explanation concerning the plots.

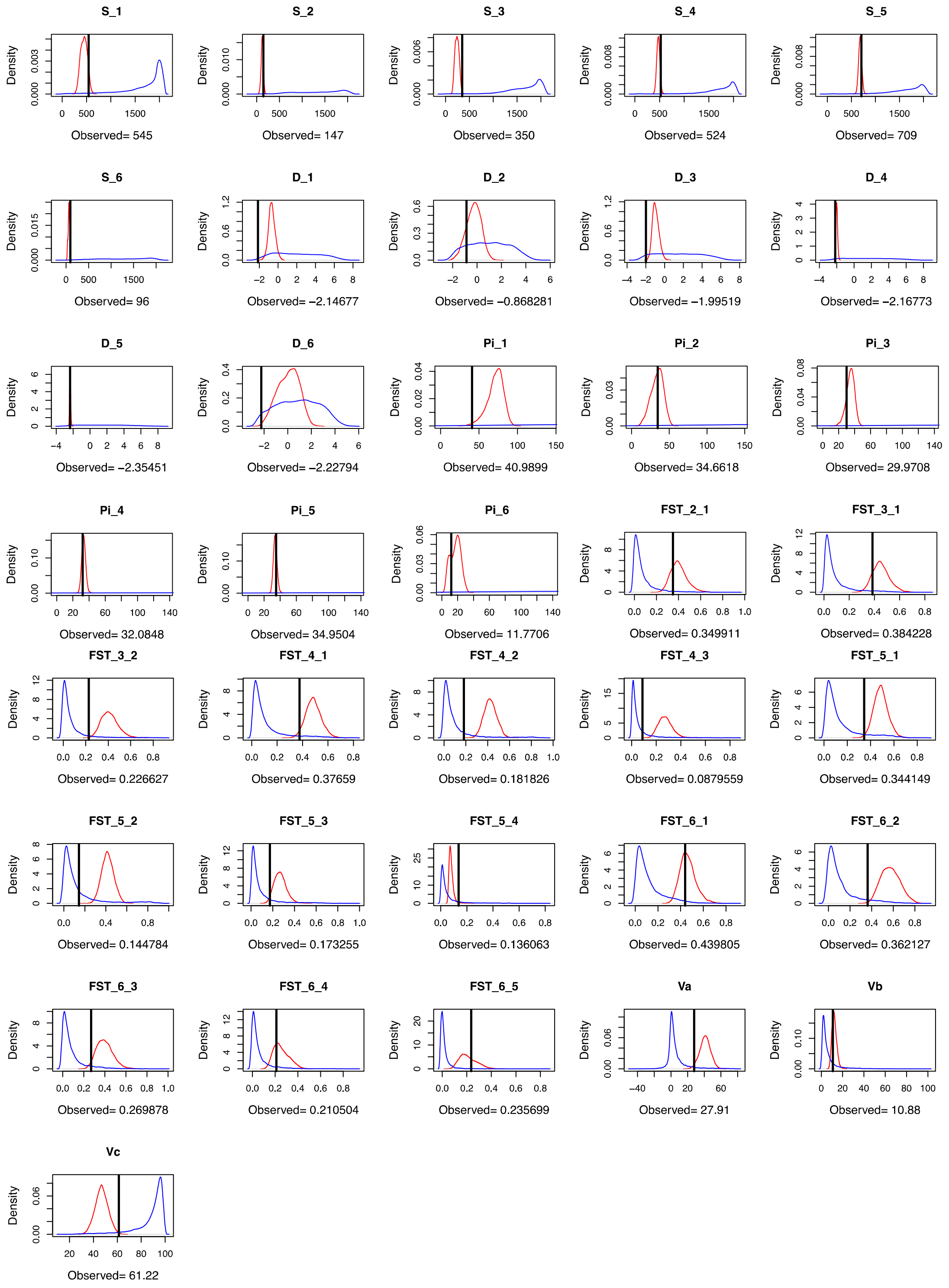

Figure S16. **Prior and posterior distributions of the summary statistics used in the simulations to estimate N_f for each regional group.** See the legend to **Figure S14** for further explanation concerning the plots.

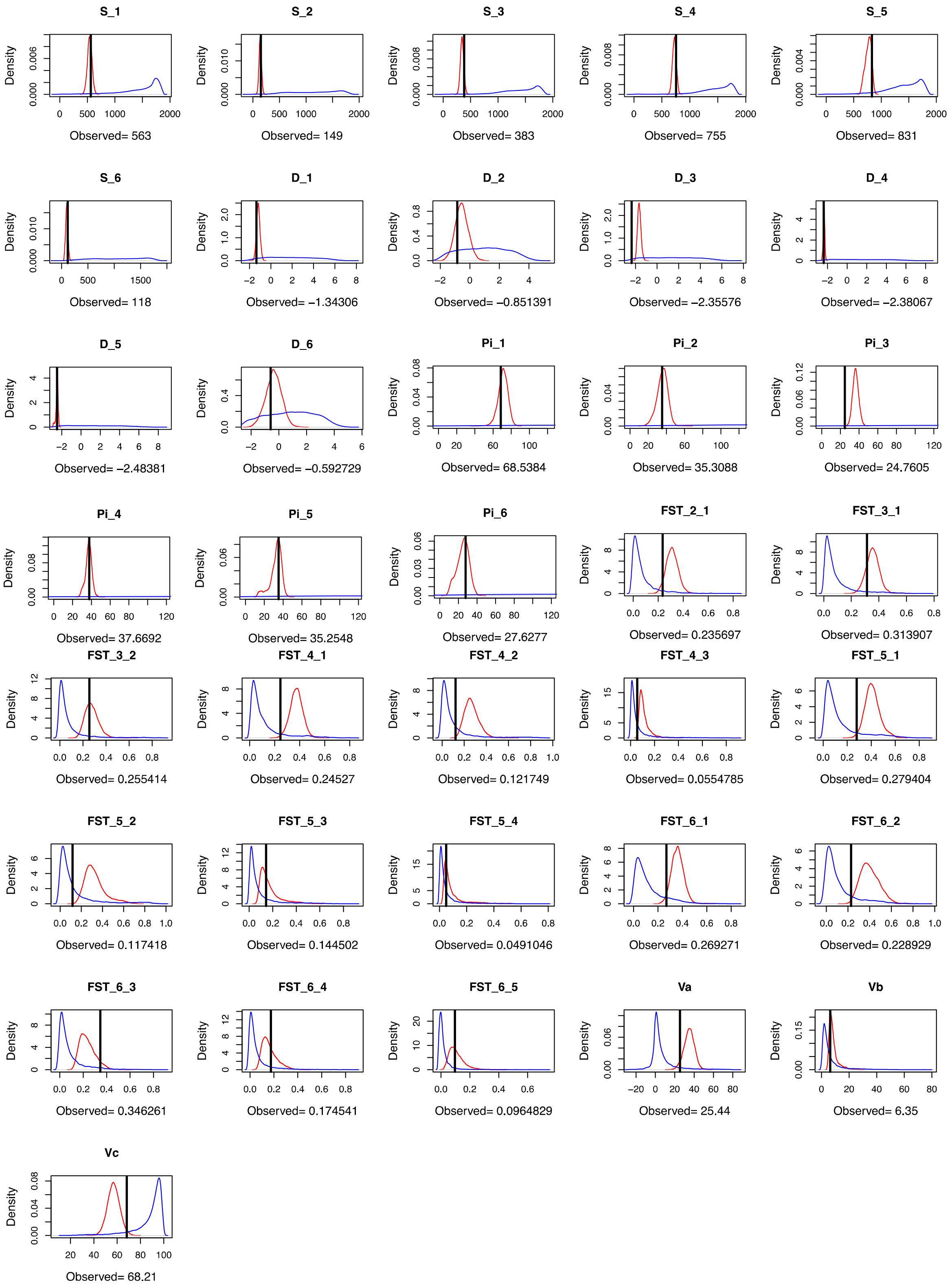

Table S3. **Summary statistics for individual HGDP populations.** (**a**) NRY sequences; (**b**) mtDNA sequences. n, sample size; h, number of different haplotypes (sequences); s, number of polymorphic sites; HD, haplotype diversity; mdp, mean number of pairwise differences; π, nucleotide diversity, SE, standard error of the respective statistic.

a) NRY sequences

Population	n	h	s	HD	HD SE	mpd	mpd SE	π	π SE
Adygei	7	6	81	0.952	0.096	33.8	16.8	0.0152	0.0087
Balochi	24	21	167	0.986	0.018	32.7	14.8	0.0147	0.0074
Bantu	18	16	198	0.987	0.023	34.8	15.9	0.0156	0.0080
Basque	16	14	98	0.975	0.035	15.3	7.2	0.0069	0.0036
Bedouin	27	19	172	0.917	0.047	28.3	12.8	0.0127	0.0064
BiakaPygmy	23	18	153	0.968	0.026	38.6	17.4	0.0173	0.0087
Brahui	25	19	211	0.967	0.024	35.4	16.0	0.0159	0.0080
Burusho	20	17	188	0.984	0.021	34.0	15.5	0.0153	0.0078
Cambodian	6	6	73	1.000	0.096	27.1	13.9	0.0121	0.0072
Colombian	2	2	13	1.000	0.500	13.0	9.5	0.0058	0.0061
Dai	7	7	107	1.000	0.076	37.2	18.5	0.0167	0.0095
Daur	7	7	124	1.000	0.076	45.6	22.6	0.0205	0.0116
Druze	12	10	129	0.970	0.044	38.7	18.1	0.0174	0.0092
French	12	12	133	1.000	0.034	34.6	16.2	0.0155	0.0082
Han	24	24	198	1.000	0.012	27.7	12.6	0.0124	0.0063
Hazara	22	13	147	0.892	0.049	36.4	16.5	0.0163	0.0083
Hezhen	6	6	99	1.000	0.096	40.9	20.8	0.0183	0.0108
Italian	7	7	62	1.000	0.076	22.1	11.1	0.0099	0.0057
Japanese	20	20	182	1.000	0.016	40.7	18.4	0.0183	0.0092
Kalash	18	8	115	0.869	0.049	33.9	15.5	0.0152	0.0078
Karitiana	6	5	14	0.933	0.122	6.9	3.8	0.0031	0.0020
Lahu	7	6	40	0.952	0.096	15.9	8.1	0.0071	0.0042
Makrani	20	19	146	0.995	0.018	29.9	13.6	0.0134	0.0068
Mandenka	15	15	97	1.000	0.024	19.2	9.0	0.0086	0.0045
Maya	2	2	12	1.000	0.500	12.0	8.8	0.0054	0.0056
MbutiPygmy	11	8	135	0.946	0.054	50.4	23.7	0.0226	0.0120
Melanesian	4	4	41	1.000	0.177	20.5	11.6	0.0092	0.0062
Miao	7	5	45	0.905	0.103	18.4	9.3	0.0083	0.0048
Mongola	7	7	117	1.000	0.076	38.5	19.1	0.0173	0.0098
Mozabite	20	11	76	0.763	0.103	16.0	7.5	0.0072	0.0037
Naxi	7	6	71	0.952	0.096	29.0	14.5	0.0130	0.0074
Orcadian	6	5	50	0.933	0.122	22.5	11.6	0.0101	0.0060
Oroqen	6	5	87	0.933	0.122	31.9	16.3	0.0143	0.0085
Palestinian	16	7	91	0.625	0.139	22.2	10.3	0.0100	0.0052
Papuan	13	12	120	0.987	0.035	37.3	17.3	0.0167	0.0088
Pathan	19	16	159	0.977	0.027	26.1	12.0	0.0117	0.0060
Pima	8	7	47	0.964	0.077	13.3	6.7	0.0059	0.0034
Russian	16	16	117	1.000	0.022	25.8	11.9	0.0116	0.0060
San	6	6	164	1.000	0.096	74.7	37.7	0.0335	0.0195
Sardinian	16	11	125	0.908	0.063	24.1	11.2	0.0108	0.0056
She	7	6	45	0.952	0.096	18.8	9.5	0.0084	0.0049
Sindhi	20	19	127	0.995	0.018	25.0	11.5	0.0112	0.0058
Surui	4	3	12	0.833	0.222	6.0	3.6	0.0027	0.0019
Tu	7	7	52	1.000	0.076	19.7	10.0	0.0088	0.0051
Tujia	9	9	126	1.000	0.052	31.2	15.1	0.0140	0.0077
Tuscan	6	6	123	1.000	0.096	45.6	23.1	0.0205	0.0120
Uygur	8	8	130	1.000	0.063	36.5	17.8	0.0164	0.0091
Xibo	8	8	119	1.000	0.063	41.7	20.3	0.0187	0.0104
Yakut	18	10	69	0.843	0.077	14.2	6.7	0.0064	0.0033
Yi	9	9	91	1.000	0.052	29.7	14.3	0.0133	0.0073
Yoruba	12	10	88	0.955	0.057	15.5	7.4	0.0069	0.0038

b) MtDNA sequences

Population	n	h	s	HD	HD SE	mpd	mpd SE	π	π SE
Adygei	7	7	121	1.000	0.076	38.5	19.1	0.0023	0.0013
Balochi	24	23	216	0.996	0.013	36.1	16.3	0.0022	0.0011
Bantu	18	16	330	0.980	0.028	75.8	34.3	0.0046	0.0023
Basque	16	16	126	1.000	0.022	24.0	11.2	0.0015	0.0008
Bedouin	27	27	425	1.000	0.010	57.8	25.7	0.0035	0.0017
BiakaPygmy	23	17	167	0.968	0.022	50.0	22.4	0.0030	0.0015
Brahui	25	22	197	0.980	0.022	33.6	15.2	0.0020	0.0010
Burusho	20	18	257	0.990	0.019	43.1	19.5	0.0026	0.0013
Cambodian	6	6	111	1.000	0.096	44.5	22.6	0.0027	0.0016
Colombian	2	2	61	1.000	0.500	61.0	43.5	0.0037	0.0037
Dai	7	7	105	1.000	0.076	40.4	20.1	0.0024	0.0014
Daur	7	7	98	1.000	0.076	31.6	15.8	0.0019	0.0011
Druze	12	11	118	0.985	0.040	24.6	11.6	0.0015	0.0008
French	12	12	123	1.000	0.034	30.3	14.3	0.0018	0.0010
Han	24	24	289	1.000	0.012	46.0	20.7	0.0028	0.0014
Hazara	22	22	265	1.000	0.014	39.4	17.8	0.0024	0.0012
Hezhen	6	6	75	1.000	0.096	33.1	16.9	0.0020	0.0012
Italian	7	7	61	1.000	0.076	20.1	10.1	0.0012	0.0007
Japanese	20	20	244	1.000	0.016	42.7	19.4	0.0026	0.0013
Kalash	18	15	125	0.974	0.029	35.6	16.3	0.0021	0.0011
Karitiana	6	5	30	0.933	0.122	12.9	6.8	0.0008	0.0005
Lahu	7	6	100	0.952	0.096	41.7	20.7	0.0025	0.0014
Makrani	20	20	279	1.000	0.016	46.9	21.2	0.0028	0.0014
Mandenka	15	12	171	0.971	0.033	56.7	26.0	0.0034	0.0018
Maya	2	2	4	1.000	0.500	4.0	3.2	0.0002	0.0003
MbutiPygmy	11	8	176	0.891	0.092	68.4	32.0	0.0041	0.0022
Melanesian	4	4	70	1.000	0.177	35.5	19.8	0.0021	0.0014
Miao	7	7	115	1.000	0.076	42.8	21.2	0.0026	0.0015
Mongola	7	7	122	1.000	0.076	43.3	21.5	0.0026	0.0015
Mozabite	20	17	174	0.979	0.025	32.4	14.7	0.0020	0.0010
Naxi	7	7	97	1.000	0.076	37.9	18.8	0.0023	0.0013
Orcadian	6	6	75	1.000	0.096	28.4	14.5	0.0017	0.0010
Oroqen	6	5	110	0.933	0.122	46.2	23.4	0.0028	0.0016
Palestinian	16	16	224	1.000	0.022	37.7	17.3	0.0023	0.0012
Papuan	13	12	141	0.987	0.035	37.0	17.2	0.0022	0.0012
Pathan	19	19	242	1.000	0.017	40.9	18.6	0.0025	0.0013
Pima	8	5	26	0.786	0.151	11.8	6.0	0.0007	0.0004
Russian	16	16	167	1.000	0.022	31.6	14.5	0.0019	0.0010
San	6	5	108	0.933	0.122	42.1	21.4	0.0025	0.0015
Sardinian	16	15	144	0.992	0.025	34.0	15.7	0.0021	0.0011
She	7	6	133	0.952	0.096	49.2	24.4	0.0030	0.0017
Sindhi	20	19	297	0.995	0.018	52.3	23.6	0.0032	0.0016
Surui	4	1	0	0.000	0.000	0.0	0.0	0.0000	0.0000
Tu	7	7	133	1.000	0.076	43.3	21.5	0.0026	0.0015
Tujia	9	9	167	1.000	0.052	47.7	22.9	0.0029	0.0016
Tuscan	6	6	75	1.000	0.096	28.0	14.3	0.0017	0.0010
Uygur	8	8	118	1.000	0.063	35.5	17.4	0.0021	0.0012
Xibo	8	8	135	1.000	0.063	43.9	21.3	0.0026	0.0015
Yakut	18	17	193	0.994	0.021	36.6	16.7	0.0022	0.0011
Yi	9	9	152	1.000	0.052	45.9	22.0	0.0028	0.0015
Yoruba	12	12	195	1.000	0.034	57.0	26.5	0.0034	0.0018

Table S4. Divergence time estimates and associated 95% CI intervals for the mtDNA and NRY phylogenies in Figure 3. In each table, Top Branch and Bottom Branch refer to the lineages that are above and below the node that is dated; “rest” refers to the remaining lineages in the phylogeny. Ages are in thousands of years.

MtDNA (Figure 3A).

Top Branch	Bottom Branch	Age	Lower 95%	Upper 95%
L0-L1	rest	159	139	174
L0	L1	148	129	163
L5	rest	134	114	149
L2	rest	107	92	120
L3-M	R-U-N	78	68	84
L3	M	73	63	80
R-U	N	69	60	73
R	U	64	56	68

NRY, fast rate (Figure 3B).

Top Branch	Bottom Branch	Age	Lower 95%	Upper 95%
rest	A	103	89	115
rest	B	77	67	86
D-E-C	rest	61	54	67
D-E	C	60	52	66
D	E	54	46	60
G-F	rest	47	40	52
G	F	41	34	46
I-J	rest	44	38	48
I	J	37	30	42
F*-H	rest	43	38	48
F*	H	39	32	45
KM	rest	39	34	43
L	rest	38	34	42
K	rest	37	32	40
O-N	rest	35	30	39
O	N	33	27	37
rest	Q	28	23	32
rest	P	27	21	31
R-R2	R1b-R1a	22	17	25
R	R2	12	7	16
R1b	R1a	19	14	22

NRY, slow rate (Figure 3C).

Top Branch	Bottom Branch	Age	Lower 95%	Upper 95%
A	rest	166	143	186
B	rest	124	108	140
C	rest	98	88	110
D-E	rest	95	85	106
D	E	86	75	98
G-F*-H-F-I-J	rest	74	65	84
G	F*-H-F-I-J	69	61	78
F*-H	F-I-J	67	59	76
F*	H	61	51	71
F	I-J	65	56	74
I	J	58	48	68
K	rest	62	55	70
P-Q-R-R2-R1b-R1a	L-K-KM-N-O	61	54	69
P	Q-R-R2-R1b-R1a	44	36	52
Q	R-R2-R1b-R1a	42	34	49
R-R2	R1b-R1a	34	27	41
R	R2	18	12	25
R1b	R1a	29	23	36
L	K-KM-N-O	59	52	65
K	KM-N-O	57	50	65
KM	N-O	56	48	65
N	O	52	44	59

Table S5. NRY haplogroup frequencies. Haplogroups were previously determined by SNP-genotyping [1, 2].

Population	A	B	C	D	E	F	G	H	I	J	K	L	M	N	O	P	Q	R
Adygei	-	-	-	-	-	-	0.43	-	-	0.29	-	-	-	-	-	-	-	0.29
Balochi	-	-	-	-	0.08	-	-	0.04	-	0.17	-	0.21	-	-	-	-	-	0.50
Bantu	0.06	0.06	-	-	0.89	-	-	-	-	-	-	-	-	-	-	-	-	-
Basque	-	-	-	-	0.06	-	-	-	-	0.06	-	-	-	-	-	-	-	0.88
Bedouin	-	0.04	-	-	0.15	-	0.04	-	-	0.70	-	-	-	-	-	-	-	0.07
BiakaPygmy	-	0.30	-	-	0.70	-	-	-	-	-	-	-	-	-	-	-	-	-
Brahui	-	-	0.04	-	-	-	0.16	0.04	-	0.28	-	0.08	-	-	0.04	-	-	0.36
Burusho	-	-	0.05	-	-	-	0.05	0.15	-	0.05	-	0.15	-	-	0.05	-	-	0.50
Cambodian	-	-	-	-	-	-	-	0.17	-	-	-	-	-	0.17	0.67	-	-	-
Colombian	-	-	-	-	-	-	-	-	-	-	-	-	-	-	-	-	1.00	-
Dai	-	-	0.29	-	-	-	-	-	-	-	-	-	-	-	0.71	-	-	-
Daur	-	-	0.29	0.14	-	-	-	-	-	-	-	-	-	0.14	0.43	-	-	-
Druze	-	-	-	-	0.08	-	-	-	-	0.42	-	0.42	-	-	-	-	-	0.08
French	-	-	-	-	0.08	0.08	-	-	0.17	0.17	-	-	-	-	-	-	-	0.50
Han	-	-	0.08	-	-	-	-	-	-	-	-	-	-	0.13	0.75	-	0.04	-
Hazara	-	-	0.36	-	-	-	-	-	0.05	0.05	-	-	-	-	0.09	0.09	-	0.36
Hezhen	-	-	0.33	-	-	-	-	-	-	-	-	-	-	-	0.67	-	-	-
Italian	-	-	-	-	-	-	0.14	-	0.14	-	-	-	-	-	-	-	-	0.71
Japanese	-	-	0.15	0.30	-	-	-	-	-	-	-	-	-	-	0.55	-	-	-
Kalash	-	-	-	-	-	-	0.17	0.17	-	0.11	-	0.28	-	-	-	-	-	0.28
Karitiana	-	-	-	-	-	-	-	-	-	-	-	-	-	-	-	-	1.00	-
Lahu	-	-	-	-	-	0.71	-	-	-	-	-	-	-	-	0.29	-	-	-
Makrani	-	-	-	-	0.05	-	-	-	-	0.25	-	0.20	-	-	-	-	0.05	0.45
Mandenka	-	-	-	-	1.00	-	-	-	-	-	-	-	-	-	-	-	-	-
Maya	-	-	-	-	-	-	-	-	-	-	-	-	-	-	-	-	1.00	-
MbutiPygmy	-	0.45	-	-	0.55	-	-	-	-	-	-	-	-	-	-	-	-	-
Melanesian	-	-	-	-	-	-	-	-	-	-	0.50	-	0.50	-	-	-	-	-
Miao	-	-	-	-	-	-	-	-	-	-	-	-	-	0.14	0.86	-	-	-
Mongola	-	-	0.14	0.14	-	-	-	-	-	-	-	-	-	0.14	0.43	-	0.14	-
Mozabite	-	-	-	-	0.90	-	-	-	-	-	-	-	-	-	-	-	-	0.10

**Population**	**A**	**B**	**C**	**D**	**E**	**F**	**G**	**H**	**I**	**J**	**K**	**L**	**M**	**N**	**O**	**P**	**Q**	**R**
Naxi	-	-	-	-	-	-	-	-	-	-	-	-	-	0.29	0.29	-	0.43	-
Orcadian	-	-	-	-	-	-	-	-	0.33	-	-	-	-	-	-	-	-	0.67
Oroqen	-	-	0.83	-	-	-	-	-	-	-	-	-	-	0.17	-	-	-	-
Palestinian	-	-	-	-	0.13	-	0.81	-	-	0.06	-	-	-	-	-	-	-	-
Papuan	-	-	0.23	-	-	-	-	-	-	-	0.62	-	0.15	-	-	-	-	-
Pathan	-	-	-	-	-	-	0.11	0.11	-	-	-	0.11	-	-	-	-	0.11	0.58
Pima	-	-	-	-	-	-	0.13	-	-	-	-	-	-	-	-	-	0.88	-
Russian	-	-	-	-	-	-	0.06	-	0.25	-	-	-	-	0.19	-	-	0.06	0.44
San	0.50	0.50	-	-	-	-	-	-	-	-	-	-	-	-	-	-	-	-
Sardinian	-	-	-	-	0.06	-	0.06	-	0.63	0.13	-	-	-	-	-	-	-	0.13
She	-	-	-	-	-	-	-	-	-	-	-	-	-	-	1.00	-	-	-
Sindhi	-	-	-	-	-	-	-	-	-	0.35	-	0.05	-	-	-	0.05	-	0.55
Surui	-	-	-	-	-	-	-	-	-	-	-	-	-	-	-	-	1.00	-
Tu	-	-	-	-	-	-	-	-	-	-	-	-	-	0.29	0.57	-	-	0.14
Tujia	-	-	0.11	-	-	-	-	-	-	-	-	-	-	0.11	0.78	-	-	-
Tuscan	-	-	-	-	0.17	-	0.17	-	0.17	0.17	-	-	-	-	-	-	-	0.33
Uygur	-	-	0.13	-	-	-	-	-	-	0.13	-	0.13	-	-	0.13	-	-	0.50
Xibo	-	-	0.38	-	-	-	-	-	-	0.13	0.13	-	-	0.13	0.25	-	-	-
Yakut	-	-	0.11	-	-	-	-	-	-	-	-	-	-	0.89	-	-	-	-
Yi	-	-	-	0.22	-	-	-	-	-	-	-	-	-	0.11	0.67	-	-	-
Yoruba	-	0.08	-	-	0.92	-	-	-	-	-	-	-	-	-	-	-	-	-

Table S6. MtDNA haplogroup frequencies. Haplogroups were inferred from the mtDNA genome sequences.

**Population**	**A**	**B**	**C**	**D**	**F**	**G**	**H**	**I**	**J**	**K**	**L**	**M**	**N**	**P**	**Q**	**R**	**T**	**U**	**V**	**W**	**X**	**Y**	**Z**
Adygei	-	-	0.14	-	-	-	0.29	-	0.14	0.14	-	-	-	-	-	0.14	-	0.14	-	-	-	-	-
Balochi	-	-	-	-	-	-	0.21	-	0.13	-	-	0.33	0.08	-	-	0.13	-	0.13	-	-	-	-	-
Bantu	-	-	-	-	-	-	-	-	-	-	1.00	-	-	-	-	-	-	-	-	-	-	-	-
Basque	-	-	-	-	-	-	0.56	-	-	0.13	-	-	-	-	-	-	0.06	0.13	0.13	-	-	-	-
Bedouin	-	-	-	-	-	-	0.22	0.04	0.07	0.07	0.26	0.04	0.07	-	-	0.11	0.04	0.04	-	-	0.04	-	-
BiakaPygmy	-	-	-	-	-	-	-	-	-	-	1.00	-	-	-	-	-	-	-	-	-	-	-	-
Brahui	-	-	-	0.04	-	-	0.28	-	0.04	-	-	0.32	-	-	-	0.04	0.12	0.16	-	-	-	-	-
Burusho	-	-	0.05	0.10	-	-	0.10	0.10	0.05	-	-	0.10	0.05	-	-	0.05	0.10	0.10	-	0.10	0.10	-	-
Cambodian	-	0.17	-	-	0.17	-	-	-	-	-	-	0.67	-	-	-	-	-	-	-	-	-	-	-
Colombian	-	0.50	0.50	-	-	-	-	-	-	-	-	-	-	-	-	-	-	-	-	-	-	-	-
Dai	-	0.14	0.43	0.14	0.14	-	-	-	-	-	-	-	-	-	-	0.14	-	-	-	-	-	-	-
Daur	-	-	0.14	0.29	-	0.29	-	-	-	-	-	0.14	-	-	-	-	-	-	-	-	-	0.14	-
Druze	-	-	-	-	-	-	0.67	-	-	0.08	-	-	-	-	-	0.17	-	0.08	-	-	-	-	-
French	-	-	-	-	-	-	0.33	-	-	0.08	-	-	-	-	-	-	0.17	0.33	0.08	-	-	-	-
Han	0.04	0.21	0.04	0.17	0.13	-	-	-	-	-	-	0.33	-	-	-	0.08	-	-	-	-	-	-	-
Hazara	-	0.05	0.05	-	0.09	0.05	0.18	-	-	-	-	0.23	-	-	-	-	-	0.27	-	-	-	-	0.09
Hezhen	-	-	0.33	0.17	-	-	-	-	-	-	-	-	-	-	-	-	-	-	-	-	-	0.50	-
Italian	-	-	-	-	-	-	0.71	-	-	-	-	-	-	-	-	-	-	0.14	0.14	-	-	-	-
Japanese	-	0.05	-	0.35	0.20	0.05	-	-	-	-	-	0.15	0.20	-	-	-	-	-	-	-	-	-	-
Kalash	-	-	-	-	-	-	-	-	0.17	-	-	-	-	-	-	0.22	0.06	0.56	-	-	-	-	-
Karitiana	-	-	-	1.00	-	-	-	-	-	-	-	-	-	-	-	-	-	-	-	-	-	-	-
Lahu	0.14	-	-	0.29	0.43	-	-	-	-	-	-	0.14	-	-	-	-	-	-	-	-	-	-	-
Makrani	-	-	-	-	-	-	0.30	-	-	-	0.35	0.20	-	-	-	0.05	-	-	-	0.05	0.05	-	-
Mandenka	-	-	-	-	-	-	-	-	-	-	1.00	-	-	-	-	-	-	-	-	-	-	-	-
Maya	1.00	-	-	-	-	-	-	-	-	-	-	-	-	-	-	-	-	-	-	-	-	-	-
MbutiPygmy	-	-	-	-	-	-	-	-	-	-	1.00	-	-	-	-	-	-	-	-	-	-	-	-
Melanesian	-	0.75	-	-	-	-	-	-	-	-	-	-	-	-	0.25	-	-	-	-	-	-	-	-
Miao	0.14	0.29	-	0.43	-	-	-	-	-	-	-	0.14	-	-	-	-	-	-	-	-	-	-	-
Mongola	0.43	0.14	0.14	0.14	-	-	-	-	-	-	-	-	-	-	-	-	-	-	-	-	-	-	0.14

**Population**	**A**	**B**	**C**	**D**	**F**	**G**	**H**	**I**	**J**	**K**	**L**	**M**	**N**	**P**	**Q**	**R**	**T**	**U**	**V**	**W**	**X**	**Y**	**Z**
Mozabite	-	-	-	-	-	-	0.30	-	0.10	-	0.15	-	-	-	-	-	-	0.35	0.10	-	-	-	-
Naxi	0.43	0.43	0.14	-	-	-	-	-	-	-	-	-	-	-	-	-	-	-	-	-	-	-	-
Orcadian	-	-	-	-	-	-	0.67	-	-	-	-	-	-	-	-	-	0.17	-	-	0.17	-	-	-
Oroqen	-	-	-	0.33	0.33	-	-	-	0.17	-	-	0.17	-	-	-	-	-	-	-	-	-	-	-
Palestinian	-	-	-	-	-	-	0.31	0.06	-	-	0.13	0.06	0.06	-	-	0.06	0.06	0.25	-	-	-	-	-
Papuan	-	-	-	-	-	-	-	-	-	-	-	-	-	0.31	0.69	-	-	-	-	-	-	-	-
Pathan	-	-	-	0.05	-	-	0.11	-	0.16	0.05	-	0.16	-	-	-	0.05	0.11	0.16	-	0.16	-	-	-
Pima	-	-	1.00	-	-	-	-	-	-	-	-	-	-	-	-	-	-	-	-	-	-	-	-
Russian	-	-	-	-	-	-	0.50	0.06	-	-	-	-	-	-	-	-	0.19	0.19	-	0.06	-	-	-
San	-	-	-	-	-	-	-	-	-	-	1.00	-	-	-	-	-	-	-	-	-	-	-	-
Sardinian	-	-	-	-	-	-	0.25	-	0.38	-	-	-	-	-	-	-	0.13	0.19	-	-	0.06	-	-
She	-	0.29	-	-	0.29	-	-	-	-	-	-	0.29	0.14	-	-	-	-	-	-	-	-	-	-
Sindhi	-	-	-	-	-	-	0.05	-	0.05	0.05	0.15	0.45	-	-	-	-	0.05	0.05	-	0.15	-	-	-
Surui	-	-	-	1.00	-	-	-	-	-	-	-	-	-	-	-	-	-	-	-	-	-	-	-
Tu	-	0.14	-	0.43	0.14	-	-	-	-	-	-	0.29	-	-	-	-	-	-	-	-	-	-	-
Tujia	0.11	0.33	0.11	0.11	0.11	-	-	-	-	-	-	0.11	-	-	-	0.11	-	-	-	-	-	-	-
Tuscan	-	-	-	-	-	-	0.67	-	-	0.17	-	-	-	-	-	-	-	0.17	-	-	-	-	-
Uygur	-	0.13	-	-	0.25	-	0.25	-	-	-	-	0.13	-	-	-	0.13	-	0.13	-	-	-	-	-
Xibo	-	-	0.38	0.13	0.13	-	-	-	-	-	-	-	-	-	-	0.13	-	0.13	-	-	-	-	0.13
Yakut	-	0.06	0.44	0.33	0.06	0.06	-	-	-	-	-	-	-	-	-	-	-	-	-	-	-	-	0.06
Yi	0.11	0.11	-	-	0.56	-	-	-	-	-	-	0.22	-	-	-	-	-	-	-	-	-	-	-
Yoruba	-	-	-	-	-	-	-	-	-	-	1.00	-	-	-	-	-	-	-	-	-	-	-	-

Table S7. **Improvement in goodness of fit of posterior vs. prior estimates of summary statistics, based on simulations to estimate population divergence times using combined mtDNA and NRY sequences.** Shown are the Bias and RMSE (defined in the legend to **Table 2**) for each summary statistic (defined in the legend to **Figure S14**) based on the prior and the posterior distributions. There is a pronounced decrease in both the bias and the RMSE in the posterior estimates, indicating that the simulations are greatly improving the fit to the summary statistics.

**Statistic**	**Observed**	**Prior**		**Posterior**	
		Bias	RMSE	Bias	RMSE
S_1	1108	2.21	2.27	-0.04	0.08
S_2	296	9.74	10.1	-0.16	0.2
S_3	733	3.82	3.88	-0.28	0.3
S_4	1279	1.81	1.84	-0.01	0.07
S_5	1540	1.3	1.34	-0.03	0.08
S_6	214	13.3	13.92	-0.08	0.15
D_1	-1.744	-3.27	-3.63	-0.4	-0.4
D_2	-0.866	-3.63	-4.24	-0.79	-0.9
D_3	-2.194	-2.57	-2.86	-0.46	-0.48
D_4	-2.301	-2.65	-2.96	-0.03	-0.04
D_5	-2.431	-2.54	-2.84	-0.02	-0.03
D_6	-1.342	-2.64	-3.03	-0.7	-0.75
Pi_1	109.528	13.41	14.78	0.35	0.37
Pi_2	69.971	20.79	22.93	0	0.16
Pi_3	54.731	25.99	28.77	0.27	0.32
Pi_4	69.754	19.52	21.8	0.06	0.11
Pi_5	70.205	18.87	21.19	0.02	0.09
Pi_6	39.398	34.32	38.41	0.24	0.31
PhiST_2_1	0.284	-0.86	0.89	0.12	0.16
PhiST_3_1	0.346	-0.84	0.88	0.06	0.1
PhiST_3_2	0.24	-0.81	0.9	0.52	0.57
PhiST_4_1	0.306	-0.77	0.84	0.23	0.25
PhiST_4_2	0.151	-0.57	1	1.25	1.28
PhiST_4_3	0.072	-0.51	1.16	1.53	1.66
PhiST_5_1	0.31	-0.73	0.82	0.26	0.28
PhiST_5_2	0.131	-0.39	1.14	1.7	1.73
PhiST_5_3	0.159	-0.7	0.9	0.23	0.37
PhiST_5_4	0.093	-0.73	0.98	-0.31	0.36
PhiST_6_1	0.343	-0.78	0.83	0.05	0.1
PhiST_6_2	0.29	-0.76	0.84	0.6	0.62

Table S8. **Improvement in goodness of fit of posterior vs. prior estimates of summary statistics, based on simulations to estimate current and ancestral N_m from the HGDP NRY sequences.** Shown are the Bias and RMSE (defined in the legend to **Table 2**) for each summary statistic (defined in the legend to **Figure S14**) based on the prior and the posterior distributions. There is a pronounced decrease in both the bias and the RMSE in the posterior estimates, indicating that the simulations are greatly improving the fit to the summary statistics.

		Prior		Posterior	
Statistics	Observed	Bias	RMSE	Bias	RMSE
S_1	545	2.14	2.28	-0.11	0.15
S_2	147	8.35	9.02	-0.05	0.14
S_3	350	3.74	3.9	-0.2	0.22
S_4	524	2.28	2.38	-0.08	0.09
S_5	709	1.33	1.43	-0.03	0.05
S_6	96	12.07	13.3	-0.28	0.31
D_1	-2.147	-1.94	-2.23	-0.57	-0.58
D_2	-0.868	-2.12	-2.85	-0.79	-0.91
D_3	-1.995	-1.78	-2.14	-0.4	-0.42
D_4	-2.168	-1.79	-2.16	-0.07	-0.08
D_5	-2.355	-1.69	-2.02	-0.01	-0.02
D_6	-2.228	-1.38	-1.59	-0.98	-1.04
Pi_1	40.990	13.12	15.32	0.72	0.75
Pi_2	34.662	14.72	17.37	0.13	0.2
Pi_3	29.971	16.44	19.48	0.23	0.26
Pi_4	32.085	14.6	17.5	0.05	0.08
Pi_5	34.950	12.48	15.26	-0.02	0.06
Pi_6	11.771	38.71	46.8	0.6	0.74
PhiST_2_1	0.350	-0.8	0.85	-0.05	0.13
PhiST_3_1	0.384	-0.77	0.82	-0.01	0.1
PhiST_3_2	0.227	-0.69	0.87	0.35	0.39
PhiST_4_1	0.377	-0.71	0.79	0.1	0.15
PhiST_4_2	0.182	-0.47	0.95	0.81	0.83
PhiST_4_3	0.088	-0.31	1.16	1.35	1.4
PhiST_5_1	0.344	-0.6	0.74	0.21	0.24
PhiST_5_2	0.145	-0.11	1.26	1.23	1.26
PhiST_5_3	0.173	-0.49	0.88	0.17	0.25
PhiST_5_4	0.136	-0.57	0.91	-0.36	0.38
PhiST_6_1	0.440	-0.73	0.78	-0.09	0.14
PhiST_6_2	0.362	-0.69	0.79	0.29	0.33
PhiST_6_3	0.270	-0.7	0.81	0.21	0.28
PhiST_6_4	0.211	-0.73	0.86	0.15	0.29
PhiST_6_5	0.236	-0.93	0.95	-0.17	0.29
Va	27.910	-0.79	0.9	0.25	0.3
Vb	10.880	-0.42	0.87	-0.01	0.15
Vc	61.220	0.43	0.48	-0.11	0.13

Table S9. **Improvement in goodness of fit of posterior vs. prior estimates of summary statistics, based on simulations to estimate current and ancestral N_f from the HGDP mtDNA sequences.** Shown are the Bias and RMSE (defined in the legend to **Table 2**) for each summary statistic (defined in the legend to **Figure S14**) based on the prior and the posterior distributions. There is a pronounced decrease in both the bias and the RMSE in the posterior estimates, indicating that the simulations are greatly improving the fit to the summary statistics.

		Prior		Posterior	
Statistics	Observed	Bias	RMSE	Bias	RMSE
S_1	563	1.6	1.74	-0.01	0.06
S_2	149	6.76	7.4	-0.01	0.12
S_3	383	2.67	2.82	-0.07	0.09
S_4	755	0.94	1.03	-0.02	0.04
S_5	831	0.69	0.8	-0.03	0.05
S_6	118	7.97	8.89	-0.07	0.15
D_1	-1.343	-2.42	-2.93	-0.08	-0.13
D_2	-0.851	-2.09	-2.79	-0.28	-0.47
D_3	-2.356	-1.62	-1.89	-0.21	-0.21
D_4	-2.381	-1.68	-1.98	-0.01	-0.02
D_5	-2.484	-1.62	-1.91	-0.02	-0.03
D_6	-0.593	-2.4	-3.77	-0.45	-0.83
Pi_1	68.538	6.11	7.31	0.04	0.07
Pi_2	35.309	11.91	14.17	0.06	0.13
Pi_3	24.761	16.66	19.82	0.34	0.35
Pi_4	37.669	10.14	12.31	0.01	0.05
Pi_5	35.255	10.22	12.61	0.02	0.07
Pi_6	27.628	13.23	16.31	0	0.14
PhiST_2_1	0.236	-0.7	0.82	0.05	0.13
PhiST_3_1	0.314	-0.71	0.79	-0.06	0.1
PhiST_3_2	0.255	-0.71	0.87	-0.09	0.16
PhiST_4_1	0.245	-0.54	0.76	0.23	0.25
PhiST_4_2	0.122	-0.2	1.27	0.64	0.69
PhiST_4_3	0.055	0.09	1.78	0.49	0.57
PhiST_5_1	0.279	-0.5	0.73	0.14	0.17
PhiST_5_2	0.117	0.11	1.56	0.89	0.93
PhiST_5_3	0.145	-0.39	0.95	-0.28	0.31
PhiST_5_4	0.049	0.17	1.96	0.04	0.3
PhiST_6_1	0.269	-0.55	0.71	0.09	0.14
PhiST_6_2	0.229	-0.5	0.8	0.38	0.43
PhiST_6_3	0.346	-0.77	0.83	-0.4	0.42
PhiST_6_4	0.175	-0.68	0.86	-0.19	0.27
PhiST_6_5	0.096	-0.81	1	0.11	0.38
Va	25.440	-0.75	0.9	0.13	0.18
Vb	6.350	-0.01	1.27	-0.02	0.14
Vc	68.210	0.28	0.34	-0.05	0.06

Supplemental References

1. de Filippo C, Barbieri C, Whitten M, Mpoloka SW, Gunnarsdottir ED, Bostoen K, Nyambe T, Beyer K, Schreiber H, de Knijff P, et al: **Y-chromosomal variation in sub-Saharan Africa: insights into the history of Niger-Congo groups.** *Mol Biol Evol* 2011, **28:**1255-1269.
2. Shi W, Ayub Q, Vermeulen M, Shao RG, Zuniga S, van der Gaag K, de Knijff P, Kayser M, Xue Y, Tyler-Smith C: **A worldwide survey of human male demographic history based on Y-SNP and Y-STR data from the HGDP-CEPH populations.** *Mol Biol Evol* 2010, **27:**385-393.
3. Underhill PA, Passarino G, Lin AA, Shen P, Mirazon Lahr M, Foley RA, Oefner PJ, Cavalli-Sforza LL: **The phylogeography of Y chromosome binary haplotypes and the origins of modern human populations.** *Ann Hum Genet* 2001, **65:**43-62.
4. Zhong H, Shi H, Qi XB, Xiao CJ, Jin L, Ma RZ, Su B: **Global distribution of Y-chromosome haplogroup C reveals the prehistoric migration routes of African exodus and early settlement in East Asia.** *J Hum Genet* 2010, **55:**428-435.
5. Scheinfeldt L, Friedlaender F, Friedlaender J, Latham K, Koki G, Karafet T, Hammer M, Lorenz J: **Unexpected NRY chromosome variation in Northern Island Melanesia.** *Mol Biol Evol* 2006, **23:**1628-1641.
6. Karafet TM, Mendez FL, Meilerman MB, Underhill PA, Zegura SL, Hammer MF: **New binary polymorphisms reshape and increase resolution of the human Y chromosomal haplogroup tree.** *Genome Res* 2008, **18:**830-838.
7. Shi H, Zhong H, Peng Y, Dong YL, Qi XB, Zhang F, Liu LF, Tan SJ, Ma RZ, Xiao CJ, et al: **Y chromosome evidence of earliest modern human settlement in East Asia and multiple origins of Tibetan and Japanese populations.** *BMC Biol* 2008, **6:**45.
8. Hammer MF, Karafet TM, Park H, Omoto K, Harihara S, Stoneking M, Horai S: **Dual origins of the Japanese: common ground for hunter-gatherer and farmer Y chromosomes.** *J Hum Genet* 2006, **51:**47-58.
9. Cinnioglu C, King R, Kivisild T, Kalfoglu E, Atasoy S, Cavalleri GL, Lillie AS, Roseman CC, Lin AA, Prince K, et al: **Excavating Y-chromosome haplotype strata in Anatolia.** *Hum Genet* 2004, **114:**127-148.
10. Semino O, Passarino G, Oefner PJ, Lin AA, Arbuzova S, Beckman LE, De Benedictis G, Francalacci P, Kouvatsi A, Limborska S, et al: **The genetic legacy of Paleolithic Homo sapiens sapiens in extant Europeans: a Y chromosome perspective.** *Science* 2000, **290:**1155-1159.
11. Francalacci P, Morelli L, Angius A, Berutti R, Reinier F, Atzeni R, Pilu R, Busonero F, Maschio A, Zara I, et al: **Low-pass DNA sequencing of 1200 Sardinians reconstructs European Y-chromosome phylogeny.** *Science* 2013, **341:**565-569.
12. Poznik GD, Henn BM, Yee MC, Sliwerska E, Euskirchen GM, Lin AA, Snyder M, Quintana-Murci L, Kidd JM, Underhill PA, Bustamante CD: **Sequencing Y chromosomes resolves discrepancy in time to common ancestor of males versus females.** *Science* 2013, **341:**562-565.
13. Sengupta S, Zhivotovsky LA, King R, Mehdi SQ, Edmonds CA, Chow CE, Lin AA, Mitra M, Sil SK, Ramesh A, et al: **Polarity and temporality of high-resolution y-chromosome distributions in India identify both indigenous and exogenous expansions and reveal minor genetic influence of Central Asian pastoralists.** *Am J Hum Genet* 2006, **78:**202-221.
14. Rootsi S, Zhivotovsky LA, Baldovic M, Kayser M, Kutuev IA, Khusainova R, Bermisheva MA, Gubina M, Fedorova SA, Ilumae AM, et al: **A counter-clockwise northern route of the Y-chromosome haplogroup N from Southeast Asia towards Europe.** *Eur J Hum Genet* 2007, **15:**204-211.
15. Pakendorf B, Novgorodov IN, Osakovskij VL, Danilova AP, Protod'jakonov AP, Stoneking M: **Investigating the effects of prehistoric migrations in Siberia: genetic variation and the origins of Yakuts.** *Hum Genet* 2006, **120:**334-353.
16. Hammer MF, Zegura SL: **The human Y chromosome haplogroup tree: Nomenclature and phylogeography of its major divisions.** *Annual Review of Anthropology* 2002, **31:**303-321.

17. O'Rourke DH, Raff JA: **The human genetic history of the Americas: the final frontier.** *Curr Biol* 2010, **20:**R202-207.
18. Sikora MJ, Colonna V, Xue Y, Tyler-Smith C: **Modeling the contrasting Neolithic male lineage expansions in Europe and Africa.** *Investig Genet* 2013, **4:**25.
19. van Oven M, Kayser M: **Updated comprehensive phylogenetic tree of global human mitochondrial DNA variation.** *Hum Mutat* 2009, **30:**E386-394.
